# Urinary chemical fingerprint left behind by repeated NSAID administration: Discovery of putative biomarkers using artificial intelligence

**DOI:** 10.1371/journal.pone.0228989

**Published:** 2020-02-13

**Authors:** Liam E. Broughton-Neiswanger, Sol M. Rivera-Velez, Martin A. Suarez, Jennifer E. Slovak, Pablo E. Piñeyro, Julianne K. Hwang, Nicolas F. Villarino

**Affiliations:** 1 Program in Individualized Medicine, Department of Veterinary Clinical Sciences, College of Veterinary Medicine, Washington State University, Pullman, WA, United States of America; 2 Department of Veterinary Clinical Sciences, College of Veterinary Medicine, Washington State University, Pullman, WA, United States of America; 3 The Animal Medical Center, New York, NY, United States of America; 4 Veterinary Diagnostic Laboratory, College of Veterinary Medicine, Iowa State University, Ames, IA, United States of America; Universitat Politecnica de Catalunya, SPAIN

## Abstract

Prediction and early detection of kidney damage induced by nonsteroidal anti-inflammatories (NSAIDs) would provide the best chances of maximizing the anti-inflammatory effects while minimizing the risk of kidney damage. Unfortunately, biomarkers for detecting NSAID-induced kidney damage in cats remain to be discovered. To identify potential urinary biomarkers for monitoring NSAID-based treatments, we applied an untargeted metabolomics approach to urine collected from cats treated repeatedly with meloxicam or saline for up to 17 days. Applying multivariate analysis, this study identified a panel of seven metabolites that discriminate meloxicam treated from saline treated cats. Combining artificial intelligence machine learning algorithms and an independent testing urinary metabolome data set from cats with meloxicam-induced kidney damage, a panel of metabolites was identified and validated. The panel of metabolites including tryptophan, tyrosine, taurine, threonic acid, pseudouridine, xylitol and lyxitol, successfully distinguish meloxicam-treated and saline-treated cats with up to 75–100% sensitivity and specificity. This panel of urinary metabolites may prove a useful and non-invasive diagnostic tool for monitoring potential NSAID induced kidney injury in feline patients and may act as the framework for identifying urine biomarkers of NSAID induced injury in other species.

## 1. Introduction

Non-steroidal anti-inflammatory drugs (NSAIDs) are massively used in both veterinary and human patients for treating pain and inflammation with approximately 111 million prescriptions dispensed each year in the U.S alone [1].

The main goal of the pharmacological control of pain and inflammation is to prevent suffering, avoid further tissue damage, and promote the recovery of normal tissue structure and organ function(s). Nevertheless, one of the greatest challenges in therapeutics is the effective and safe long-term use of NSAIDs for conditions requiring sustained anti-inflammatory or pain relief because the repeated administration of NSAIDS can result in gastrointestinal and kidney damage [2], [3], [4].

Early stages of kidney injury are often subclinical. If kidney damage following acute kidney injury (AKI) is not appropriately repaired with normal renal parenchyma, this can lead to chronic kidney disease (CKD) [5]. NSAID-induced kidney damage severely limits the possibility to provide optimal treatment for a variety of conditions, including: degenerative joint disease, cancer, etc., all of which require long-term anti-inflammatory therapy.

A potential contributing factor to the development of kidney disease is delayed detection due to the lack of non-invasive and sensitive diagnostic tests. The most commonly utilized clinical diagnostic tests for detection and monitoring of AKI and CKD include serum biochemical values including blood urea nitrogen (BUN), creatinine and urine concentrating ability. However, these tests have severe limitations which hamper early detection and diagnosis of kidney damage [6, 7]. Unfortunately, serum creatinine and BUN are late markers of kidney filtration and function, with limited specificity and impaired capacity to accurately reflect the timing and severity of injury.

The European Medicines Agency and the US Food and Drug Administration have encouraged further development of eight biomarkers of drug-induced-kidney-injury in early clinical trials (EMEA/FDA guideline on detection of drug-induced nephrotoxicity. 2009). To date, however, no specific biomarkers have been identified to detect early kidney damage induced by NSAIDs. A single-biomarker-to-single-disease approach is highly unlikely to capture the multifactorial nature of a dynamic progressive process such as kidney disease; whereas a panel of biomarkers would increase the likelihood of detecting early subclinical kidney damage.

Metabolomics can be applied to many body fluids, such as urine and blood, to identify alterations in concentrations of 0.8–1.2 kDa molecules. Altered metabolites act as a chemical fingerprint and can then potentially be utilized as biomarkers for detecting or monitoring biological processes [8, 9]. Urine is commonly used to study diseases of the renal system because it offers advantages owing to its minimal invasiveness and non–volume-limited nature. In addition, urine can be used for measuring time-related metabolic alterations—a valuable feature for the study of progression and prognostication of acute and chronic diseases [10]. Urine metabolomics has been successfully applied to understand the mechanism(s) underlying drug toxicity and discover novel biomarkers for detecting kidney disease [11].

The repeated administration of NSAIDs in cats is known to damage kidneys [12]. Therefore we hypothesize that the repeated administration of meloxicam alters the urine metabolome. We addressed this hypothesis by comparing the urine metabolome in meloxicam- and saline treated cats. Considering that the metabolite profile is analogous to a chemical fingerprint left behind by NSAID-induced alterations in cellular processes another objective of this study is to identify small molecular weight substances in urine that could be further studied as putative biomarkers for monitoring the administration of NSAIDs. Identification of putative biomarkers for monitoring the administration of NSAIDs was accomplished by applying multivariate analysis and artificial intelligence machine learning algorithms.

Artificial intelligence is a rapidly expanding specialty within the field of computer science. Current uses for artificial intelligence include prediction of Amazon shopping preferences, search engine recommendations, the training of Google self-driving cars and prediction of patient outcomes from medical ailments such as myocardial infarction [13]. Machine learning is a subbranch of artificial intelligence in which patterns and statistical models are extracted from large complex data sets to train algorithms to identify potentially complex patterns and interactions within large data sets (e.g. metabolomics data) [14, 15]. These learned patterns can be applied to test data sets of interest to make outcome predictions. In the present study, we trained machine learning algorithms on an initial urine metabolomics training data set to make data driven identification of putative urinary biomarkers for monitoring cats treated repeatedly with an anti-inflammatory dose of meloxicam. Then, the panel of metabolites was validated using an independent testing urinary metabolome data set from cats with meloxicam-induced kidney damage.

## 2. Materials and methods

The Washington State University Institutional Animal Care and Use Committee approved all study procedures before their initiation (ASAF#04915 and #04662). All methods were carried out in accordance with the relevant guidelines and regulations.

### 2.1 Study population and inclusion criteria

For the biomarker discovery experiment, a training data set of 12 female clinically healthy intact adult (1–1.5 years old) purpose bred cats (2.5–3.8 kg) were obtained from a USDA-licensed commercial breeder (Nutrition and pet Care Center UC DAVIS, Davis, USA).

For the biomarker validation experiment, a testing data set of 8 female clinically healthy intact adult (1–2 years old) cats (2.8–3.7 kg) were obtained from a USDA-licensed commercial breeder (Liberty Research, Inc., Waverly, NY).

The enrollment criteria for both training and testing data set cats were no signs of illness or infection and no history of drug treatment within 30 days prior to initiation of the study. Each cat enrolled in the study was given a baseline complete history, physical examination, and laboratory evaluation. All cats had normal complete blood count, serum chemistry, and urinalysis. All cats were vaccinated for rabies prior to transfer to Washington State University.

#### 2.1.1 Animal management and monitoring

Cats from both groups were acclimated to the new housing environment at least 10 days before beginning the study. Each training data set cat was housed separately in cages 49” wide, 37” tall and 38” deep. Cages were set up in the room in a way compatible cats could see one another. Each testing data set cat was housed separately in a 4’ X 8’ partitioned room. A see-through barrier partitioned the room so the cats could see each other. Bedding, cat trees, and additional enrichment, including toys, were provided to all cats.

For both groups of cats, the room was temperature (21–23°C), humidity (25–35%) and 12h light/dark cycle controlled. The cats had free access to drinking water and food (Purina Cat Chow Indoor Formula) throughout the study. Fresh water and food were changed every day. Cages/rooms were cleaned, and litter boxes changed every day. Cats were examined at least twice daily during the entire study to rule out possible health problems. Body weight and condition scores were assessed every 2 days during the duration of the study.

Following the acclimation period, vascular access ports (VAP, petite size) (Le Port Companion Port, Norfolk Vet, Skokie, IL, USA) were implanted subcutaneously in the training data set of cats (n = 12) at least 7 days before starting the administration of the treatments following standard procedures as recommended by the manufacturer. VAPs were implanted aseptically in the jugular vein. The aspiration port was implanted subcutaneously in the dorsal aspect of the neck cranial to the interscapular space. The VAPs were maintained with heparinized saline solution (100 I.U./mL, 0.7 mL each time) following the manufacturer recommendations.

A long-term indwelling jugular catheter (Mila International, Inc) was placed in each testing data set of cats at least 1 day before starting the study. The jugular catheters were maintained with 0.5 mL of heparinized saline solution (10 IU/mL, 2–4 times a day).

#### 2.1.2 Controlled randomized experimental design

Following the VAPs implantation, the training data cats were randomly allocated to two groups; a saline control group (n = 6) and a meloxicam treatment group (n = 6). For the testing data set cats, following the acclimation period, cats were allocated randomly to control (n = 4) and meloxicam (n = 4) groups. Randomization of the treatments for both the training and testing set was done using simple randomization in R studio. At the end of the treatment phase, three cats from each training data set group were euthanized within 24h after the last treatment. The remaining cats in the training data set group (n = 3 per group) were monitored for up to 16 days after the last administration of treatments and were euthanized within 24h after the last monitoring day. All testing data set cats (n = 8) were euthanized within 24h of the last treatment. An intravenous overdose of pentobarbital (Beuthanasia-D, Intervet/Merck Animal Health, Giralda Farms, Madison, NJ) was used for euthanasia of all cats.

#### 2.1.3 Treatment administrations

Prior to initiation of the biomarker discovery experiment, training data set cats (n = 12) were treated subcutaneously with 0.1 mL/kg body weight of saline every 24h on days -3, -2 and -1 (6 p.m. +/- 1h). Beginning on day zero, cats in the meloxicam group were treated subcutaneously with meloxicam at a dosage of 0.3 mg meloxicam/kg body weight (equivalent to 0.1 mL/kg) (Metacam® injectable, Boehringer Ingelheim Vetmedica, *inc*) every 24h for 31 days. Cats in the control group received 0.1 mL/kg body weight of saline subcutaneously every 24h. The duration of these treatments was defined based on preliminary experiment information.

For the biomarker validation experiment, testing data set cats in the control group were treated subcutaneously with a volume of saline at 0.1 ml/kg of body weight (equivalent volume) every 24 h (7 a.m. +/- 2 h) for 17 days. Testing data set cats were treated with 0.3 mg meloxicam/kg body weight (Metacam® Injectable, Boehringer Ingelheim Vetmedica, Inc) every 24 h (7 a.m. +/- 1 h) for 17 days.

#### 2.1.4 Blood, plasma and urine collection

Blood and urine samples were collected on days -3, -1, 4, 9, 13, 17, 23, 26, 31, 34, 40, and 47 for the training data set of cats and on days -1, 2, 13 and 17 for the testing data set of cats. For both sets of cats, food was withheld 8h prior to collection of blood and urine samples. Blood and urine samples were collected during the afternoon (7 p.m. +/- 1h). Blood samples were collected into 3.2% sodium citrate tubes and kept in ice until centrifugation. Plasma samples were collected by centrifugation (1800 x g during 8 min) within one hour upon blood sample collection. Urine samples were collected by ultrasound-guided cystocentesis following standard procedures. Upon urine collection, approximately 100 μL of urine was used to estimate the urinary specific gravity. The remaining volume of the urine sample was maintained in ice and was centrifuged at 1800 x *g* for 8 min within one hour upon collection. The supernatant was aliquoted and stored at -80°C until metabolomic analysis. Urine metabolomic analysis was done within 6 months of sample collection. One cat in the treatment group of the testing data set of cats (M4) was euthanized early due to declining health and samples were collected on days 4, 9, 11 for this cat. The urine samples collected for cat M4 on days 9 and 11 were included in the metabolomics statistical analysis for days 13 and 17, respectively.

#### 2.1.5 Creatinine quantification and urine specific gravity (USG) determination

For both the training and testing data set urine samples, plasma creatinine concentrations were measured using the QuantiChrom TM Creatinine Assay Kit (BioAssay Systems, Hayward, CA). Urinary specific gravity was determined at room temperature within 2h upon sample collection using a refractometry.

#### 2.1.6 Kidney histopathology

Immediately following euthanasia, cross sections of the left and right kidney, encompassing the capsule, cortex, and medulla, from each animal from both the training and testing data sets were trimmed to a maximum thickness of 0.5 cm and fixed in 10% formaldehyde. Paraffin-embedded kidney specimens were cut at a 5 μm thickness and stained with either hematoxylin and eosin (H&E), Masson’s trichrome, or periodic acid-Schiff methenamine silver (PAMS) stains. Each of the three stains was performed on each kidney sample. Histologic changes of the renal tissues were scored by a blinded pathologist using a modified semiquantitative scoring system [16]. A representative section of at least one kidney from each testing data set animal was scored. Traits related to tubular-interstitial disease, including cortical and medullary tubular damage, basement membrane integrity, interstitial fibrosis, inflammation and if present, the distribution of the inflammation were evaluated counting 50 cortical and medullary tubules from at least 10 different fields for each animal. A semiquantitative scoring system was used as follows: 0, no disease; 1, 1–25%; 2, 26–50%; 3, 51–75%; and 4, 76–100% of tissue affected. Higher scores represent more severe histologic changes.

### 2.2 Urine metabolomics

#### 2.2.1 Metabolite determination

**Fig 1:**
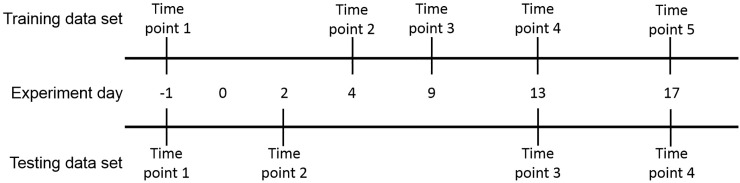
Sampling schedule for metabolite determination in the training and testing groups. Day -1 represents the pretreatment time point. For both the training and testing data sets, starting on day 0 cats in the treatment groups for each set received 0.3 mg/kg meloxicam every 24h till the end of the study. Cats in the control groups received 0.1 mL/kg body weight of saline every 24h until the studies end.

Metabolomics analyses for the training and testing data sets were performed at the West Coast Metabolomics Center (Davis, CA), a National Institute of Health Regional resource core. For the training data set, metabolomics was performed on urine samples collected from meloxicam- and saline-treated cats day -1 (time point 1) and on days 4, 9, 13, and 17 (time points 2–5, respectively). For the testing data set, metabolomics was performed on urine samples from control and treated cats collected on day -1 (time point 1) and on days 2, 13, and 17 (time points 2–4, respectively). A graphic representation of the sampling time line is provided in Fig 1. The methods used to determine the urine feline metabolome was fully described elsewhere [17, 18] and described briefly as follows.

#### 2.2.2 Metabolite identification

The metabolomics data acquisition for feline plasma and urine samples was performed at the West Coast Metabolomics Center (Davis, California), a National Institute of Health regional resource laboratory on May 2017 for the testing data set samples and March 2016 for the training data set samples. An in-depth description of the methods utilized have been previously published and are summarized below ([17], [19], [20]).

*2*.*2*.*2*.*1 Extraction*, *clean-up and derivatization*. A 30 μL aliquot of urine sample was added to a 1.0 mL cooled extraction solution composed of acetonitrile and isopropanol (JT Baker Center Valley, PA, USA) and 18.2 MΩ water (MilliQ) at a 3:3:2 v/v/v ratio in a 1.5 mL polypropylene tube. Before adding the extraction mixture to the study samples, oxygen was removed by sparging nitrogen for 5 minutes. Internal standards with isotope-labeled metabolite surrogates were added to the study samples. The study samples were mixed by vortexing for 10 s and then placed in a chilled (4°C) shaker for 5 mins. Samples were then centrifuged at 14,000 rcf for 2 minutes to pellet insoluble material. A 450 μL aliquot of the supernatant was removed, placed in a fresh 1.5 mL polypropylene tube and evaporated using a cold trap concentrator. The dried samples were re-suspended with 450 μL of 50% acetonitrile and centrifuged at 14,000 rcf for 2 minutes. The supernatant was transferred to a new tube and desiccated using a cold trap concentrator. The dried samples were derivatized by adding 10 μL of 40 mg/mL of methoxyamine hydrochloride (Aldrich, St. Louis, MO, USA) and shaken at maximum speed at room temp 30°C for 1.5 h. A 10 μl aliquot of fatty acid methyl esters (FAME, recipe provided in Fiehn 2017) was added to 1 mL of N-methyl-N-(trimethylsilyl)-trifluoroactamide (MSTFA: Aldrich, St. Louis, MO, USA) and vortexed for 10 s. Finally, 91 μL of the MSTFA + FAME mixture was added to each sample at shaken at 37°C at maximum speed for 30 minutes. The final mixture was transferred to glass vials containing micro-inserts and immediately sealed.

A reagent and derivatization blank was used per batch of 50 sample extractions as negative control. A reagent blank uses all reagents, but does not use extraction tubes or extraction solvents.

*2*.*2*.*2*.*2 Chromatography and mass spectrometry data acquisition*. Samples were separated using an RTX-5Sil MS analytical column made of 0.25 μm thick 95% dimethyl/5%diphenylpolysiloxane film which is 30 m long with a 0.25 mm internal diameter (Restek corporation, Bellefonte, PA USA). This analytical column is surrounded and protected by a 10 m long guard column. The mobile phase consisted of pure helium with a flow-rate of 1 mL/min and a column temperature of 50–330°C. The sample injection volume was 0.5 μL at 50°C ramped to 250°C by 12°C/s. The oven was held at 50°C for 1 minute and then ramped to 330°C and held for 5 minutes. The ramp speed for the oven is 20°C/min. An Agilent 6890 gas chromatograph with a Leco Pegasus IV gas chromatography, time-of-flight mass spectrophotometer (GC-TOF-MS) controlled with Leco ChromaTOF software version 2.32 were used for metabolite data acquisition. The mass spectrophotometer parameters included unit mass resolution at 17 spectra/s from 80–500 Da at -70 eV ionization energy and 1800 V detector voltage with a 230°C transfer line and a 250°C ion source.

*2*.*2*.*2*.*3 Spectra data processing and metabolite identification*. Raw data files were preprocessed using ChromaTOF version 2.32 with the following settings: no smoothing, 3 s peak width, baseline subtraction, automatic spectral deconvolution and peak detection at signal/noise levels of 5:1 throughout the chromatogram. Spectra which failed to meet at least one of these parameters were considered not identified for purposes of this study. All spectra identification was done using the BinBase database.

The BinBase algorithm (rtx5) used the following settings: validity of chromatogram (10^7 counts s -1), unbiased retention index marker detection (MS similarity>800, validity of intensity range for high m/z marker ions), retention index calculation by 5th order polynomial regression. Spectra were cut to 5% base peak abundance and matched to database entries from most to least abundant spectra using the following matching filters: Parameters used to identify spectra included retention index window ± 2000 U (around ± 2 s retention time deviation), unique ion and apex mass validation, mass spectrum similarity and a final isomer filter. Peak height was reported as quantification using the unique ion as default. A quantification report table was produced for all database entries that are positively detected in more than 10% of the samples of a study design class for unidentified metabolites. A subsequent post-processing module was employed to automatically replace missing values. Replaced values were labeled as ‘low confidence’ by color coding, and for each metabolite, the number of high-confidence peak detections was recorded as well as the ratio of the average height of replaced values to high-confidence peak detections. These ratios and numbers were used for manual curation of automatic report data sets to data sets released for submission. Peak heights (mz value) at the specific retention index were reported. Peak heights instead of peak areas because peak heights are more precise for low abundant metabolites than peak areas, due to the larger influence of baseline determinations on areas compared to peak heights.

#### 2.2.3 Data processing

All metabolomics data analyses carried out in this study were performed using Metaboanalyst 4.0, an open source R-based program specifically designed for metabolomics [21]. The raw data was filtered using standard deviation to identify and remove variables which were non-informative to downstream modeling [22]. Additionally, the raw data was normalized through log (base 2) generalized transformation to the metabolite peak intensities to stabilize the variance and autoscaled by mean-centering and dividing by the standard deviation of each variable [23], [24].

#### 2.2.4 Identification of putative biomarkers using a training metabolome data set

In order to minimize the likelihood of capturing irrelevant metabolites, we applied in tandem three statistical approaches to the training data set including partial least squares discriminant analysis (PLS-DA) variable importance of projection scores (VIP), random forest (RF) mean decrease in accuracy (MDA) values and, univariate receiver operating curve (ROC) area-under the curve (AUC) analysis to the complete set of identified metabolites, for each time point separately.

A combination of four selection criteria were used to identify metabolites as potential biomarkers in the training data set for discriminating meloxicam-treated from saline-treated cats. These criteria include metabolites with an i) VIP >1, ii) MDA > 0.4%, iii) AUC >0.85, in at least one treatment time point and iv) only metabolites which were identified at each treatment time point [25], [26], [27]. Metabolites which met these inclusion criteria were considered putative biomarkers and selected for additional analyses in the testing data set.

#### 2.2.5 Supervised dimension reduction

PLS-DA was applied to each time point, 1 through 5, individually to compare meloxicam treated cats against the control groups for supervised dimension reduction and initial identification of phenotypically significant urine metabolites of acute feline kidney injury. VIP values were used as one method of identifying biomarkers with predictive value in separating meloxicam treated cats from control cats. Metabolites with VIP scores ≤1 at each time point were considered irrelevant to the prediction and excluded from analysis [25], [28]. The predictive ability of the model for each time point was internally validated based on leave-one-out cross validation using the Q2 diagnostic statistic [29].

#### 2.2.6 Random forest classification

The supervised machine learning random forest algorithm was applied to cluster cats based on their phenotype (meloxicam and saline treatments) and classify metabolites according with their contributions to classification accuracy. The parameters used for the analysis included 500 trees, 7 predictors and randomness. Each tree attempts to identify metabolites which contribute the most information to discriminate treatment from controls. Those metabolites with >0.4% MDA in the random forest analysis during at least one treatment time point were considered for inclusion in the model [26].

#### 2.2.7 Receiver operating characteristic curve analysis

Univariate ROC curve analysis was used to identify molecules that may represent candidate biomarkers for detecting changes in urine induced by the repeated administration of meloxicam. We initially used this approach by assessing the complete set of identified urine metabolites between the saline and meloxicam group at each time point. The area under the ROC curve (AUC) value was used as a measure of the prognostic accuracy of individual metabolites. An AUC value of 1.0 indicates a perfect test due to absence of overlap of the test data from the control and diseased states, whereas an AUC value of 0.5 shows the test is no better than random chance and therefore has no diagnostic or prognostic value. We only considered metabolites with an AUC >0.85 for inclusion in the model [27].

#### 2.2.8 Validation of putative biomarkers using a separate testing metabolome data set

The putative biomarker model was trained on training data set cats and then applied to a separate testing urine metabolite data set generated from an independent experiment of cats (n = 8).The select set of biomarkers was utilized in the test data set to perform multivariate ROC curve analysis with PLS-DA and RF machine learning, separately, as the classification and feature ranking method performed. Sensitivity and specificity, ROC curves, Monte Carlo cross-validation confusion matrices, predictive accuracies and mean importance were calculated for each testing data set time point to validate the value of the metabolite model derived from the training data set. [30], [31].

## 3. Results

### 3.1 Training metabolome data set

All animals included in this study were clinically healthy according to physical examinations, cellular blood counts and blood chemistry panels prior to administration of treatments. Once treatments were started, cats in the control group remained healthy for the duration of the study. One cat in the control group vomited once on day 4 after saline administration. In the meloxicam treated group, the cats’ body-weights and condition scores were relatively stable, with the exception of one cat (M5) whose body weight was reduced by ~ 7%, likely due to a decrease in food intake. During the period of sample collection (0–17 days of treatments), 5 out of 6 cats in the meloxicam group vomited 2 to 15 times but no more than once a day. However, their food intake, body weight, and condition scores were consistent with pre-treatment values. One cat in the training experiment, meloxicam-treated group failed to develop clinical pathologic signs of AKI kidney damage (changes in serum creatinine concentrations and USG) during the study and lacked histopathologic lesions consistent with kidney injury. This cat had comparable plasma concentrations of meloxicam as compared to other cats within the meloxicam treatment group (unpublished data). Due to the lack of clinical kidney disease, this single cat was censored from the biomarker discovery analysis.

### 3.2 Training data set creatinine and USG

Serum creatinine concentrations initially rose in the meloxicam treated group on day 9 and stayed markedly elevated for the remainder of the study, consistent with a decreased glomerular filtration rate (S1 Fig). Similarly, the USG began to drop on day 9 within the meloxicam treated group, consistent with perturbations in concentrating ability secondary to renal tubular damage (S2 Fig).

### 3.3 Urine metabolome of training data set

By applying an untargeted metabolomics approach, we were able to detect 318 low molecular-weight (LMW) substances in the urine. Of these, 114 have been identified (Table 1), while 204 remain unknown (when spectra or the retention index is not matched to library entries).

**Table 1:** List of 114 low molecular-weight molecules identified in feline urine in the training data set using an untargeted metabolomics approach.

3-(3-hydroxyphenyl)propionic acid	glycerol	phenaceturic acid
3-(4-hydroxyphenyl)propionic acid	glycerol-3-galactoside	phenol
3,4-dihydroxycinnamic acid	glycine	phosphate
3,4-dihydroxyhydrocinnamic acid NIST	glycocyamine	pimelic acid
3,4-dihydroxyphenylacetic acid	glycolic acid	pinitol
3-aminoisobutyric acid	hexadecane	propane-1,3-diol NIST
3-hydroxy-3-methylglutaric acid	hexitol	pseudo uridine
3-phosphoglycerate	hexuronic acid	putrescine
4-hydroxybenzoate	hippuric acid	pyruvic acid
4-hydroxybutyric acid	hydroxylamine	quinic acid
4-hydroxycinnamic acid	indole-3-acetate	raffinose
4-hydroxyhippuric acid NIST	indoxyl sulfate	ribitol
4-hydroxyphenylacetic acid	inosine	ribonic acid
5-hydroxy-3-indoleacetic acid	isocitric acid	ribose
aconitic acid	isohexonic acid	saccharic acid
adenosine	isomaltose	serine
alanine	isoribose	sorbitol
allantoic acid	isothreonic acid	stearic acid
alpha-ketoglutarate	kynurenic acid	succinic acid
benzoic acid	lactic acid	sucrose
benzylalcohol	lysine	sulfuric acid
beta-alanine	lyxitol	tagatose
beta-gentiobiose	lyxose	taurine
catechol	malic acid	threitol
citramalic acid	mannose	threonic acid
citric acid	mucic acid	trehalose
citrulline	myo-inositol	tryptophan
conduritol-beta-expoxide	myristic acid	tyrosine
creatinine	N-acetylaspartic acid	tyrosol
deoxypentitol	n-acetyl-d-hexosamine	urea
erythritol	N-acetylglutamate	uric acid
ferulic acid	N-acetylmannosamine	uridine
fructose	ornithine	valine
fucose	oxalic acid	vanillic acid
galactinol	oxoproline	xylitol
galactonic acid	palmitic acid	xylonic acid
gluconic acid	pelargonic acid	xylose
glyceric acid	pentitol	xylulose NIST

### 3.4 Identification of putative biomarkers using a training metabolome data set

#### 3.4.1 Dimension reduction and cluster analysis

**Fig 2:**
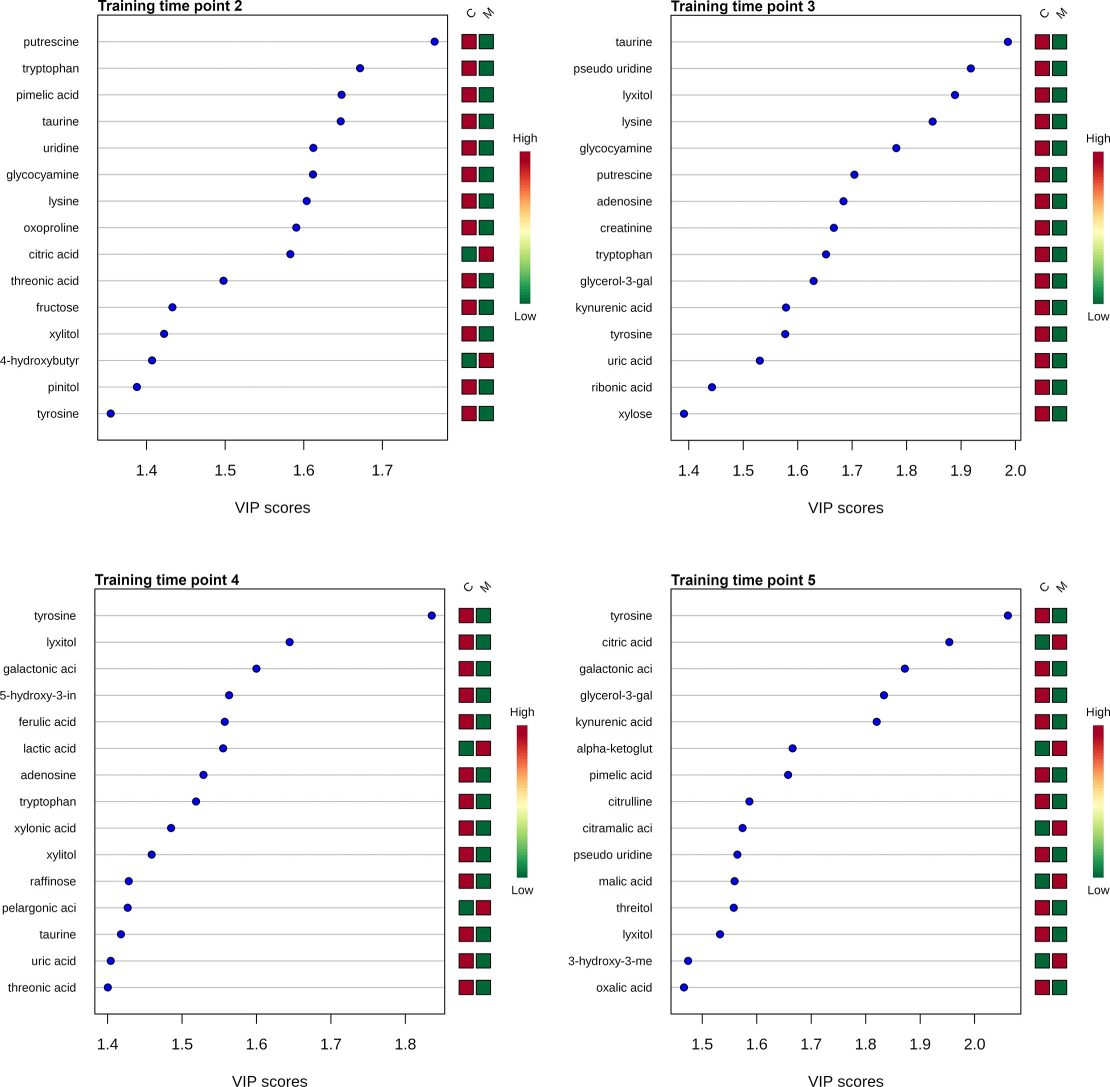
Variable importance in projection (VIP) scores for the top 15 metabolites. VIP scores of top 15 urine metabolites used to differentiate meloxicam-treated (n = 5) and saline-treated cats (n = 6). VIP scores are derived from PLS-DA analysis performed at each time point, 1–5. VIP scores ≥ 1.0 were considered significant when selecting metabolites for the final model. The column to the right of each figure display variations in metabolite peak intensities.

PLS-DA was efficient at discriminating between groups as these supervised multivariate techniques take group identity (i.e. treatment vs control) into account during dimension reduction analysis. The Q2 statistics of performance, an estimate of the predictive ability of the discriminant analysis model, for time points 1 through 5 were 0.03, 0.55, 0.52, 0.52, and 0.33, respectively. These Q2 values support that there was not overfitting of the data, except for at time point 1 (prior to meloxicam administrations). However, this is expected for time point 1 as PLS-DA will attempt to force a distinction utilizing group identity, even prior to treatment (overfitting). The top 15 VIP results for each time point of the PLS-DA are presented in Fig 2. VIP scores summarize the influence of an individual metabolite on the overall PLS-DA model (S1 Table). VIP scores ≥ 1 indicate metabolites which contribute significantly to the model.

#### 3.4.2 Random forest classification

**Fig 3:**
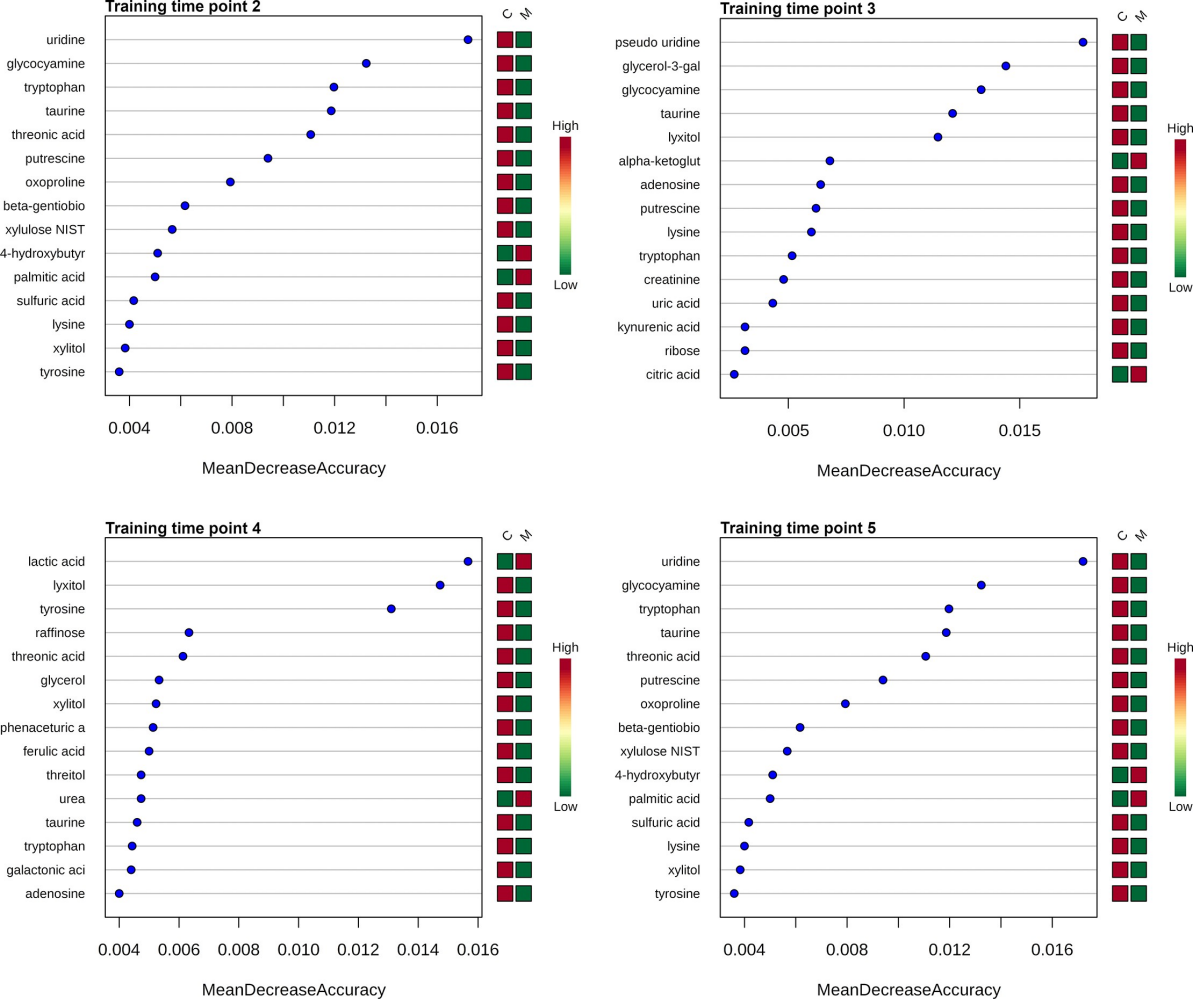
Mean decrease in accuracy (MDA) of top 15 urine metabolites. MDA of the top 15 urine metabolites for the discrimination of meloxicam-treated (n = 5) and saline-controls (n = 6) for each time points (2–5) after the administration of meloxicam derived from random forest analysis. The higher the MDA value the more important a metabolite is to the performance of the model. Urine metabolites with a mean decrease in accuracy ≥ 0.004 in at least one post-treatment time point (2–5) were considered for inclusion in the predictive model.

Metabolites for time points 2–5 were ranked using machine learning algorithms by contributions to the performance of the model as measured by the MDA value (Fig 3). A higher MDA value indicate increasing importance of a metabolite in predicting phenotype group (control vs. meloxicam). There are 15, 14, 10 and 8 metabolites which met this inclusion criteria at time points, 2, 3, 4 and 5, respectively during the RF classification step (S2 Table).

#### 3.4.3 Receiver operating characteristic curve analysis

Univariate ROC AUC values were calculated for all metabolites. AUC values represent the accuracy of an individual metabolite in correctly separating treatment from control animals. Prior to administration of treatments (time 1), 7 metabolites had an AUC > 0.85 including citrulline, alpha-ketoglutarate, tyrosol, mucic acid, citric acid, mannose and 3-hydroxy-3-methylglutaric acid. These seven metabolites were excluded from the training data set time points 2–5 for all analyses performed on the training data set i.e PLS-DA and RF analyses (S3 Table).

#### 3.4.4 Training data set putative biomarkers

Metabolites present at all treatment time points which met the inclusion criteria of AUC ROC > 0.85, VIP score > 1.0 and an MDA > 0.004 in at least one time point include tyrosine, taurine, tryptophan, pseudouridine, lyxitol, xylitol and threonic acid (Table 2). These seven metabolites were selected for inclusion in the model as potential biomarker candidates. To evaluate the performance of the proposed seven metabolite model, multivariate ROC analysis was performed on each treatment time point, 2–5, and results were verified by the Monte Carlo cross-validation method. The sensitivity and specificity of the seven metabolite model based on either PLS-DA or RF as the final classifier are listed in Table 3 when applied to the training data set.

**Table 2:** List of putative urine biomarkers derived from the training data set. The selected seven urine metabolites were the only compounds in this study which met the following inclusion criteria: MDA ≥ 0.004, VIP score > 1.0, ROC AUC > 0.85 and in at least one treatment time-point.

**Time 2**	**MDA**	**VIP score**	**ROC AUC**
Tryptophan	1.20%	1.7	1.0
Taurine	1.19%	1.6	1.0
Threonic acid	1.11%	1.5	1.0
Tyrosine	0.36%	1.4	0.93
Lyxitol	0.23%	1.4	0.93
Xylitol	0.38%	1.4	0.93
Pseudouridine	0.35%	1.3	0.9
**Time 3**	**MDA**	**VIP score**	**ROC AUC**
Tryptophan	0.52%	1.6	0.9
Taurine	1.21%	2.0	1.0
Threonic acid	0.00%	1.3	0.9
Tyrosine	0.17%	1.6	0.93
Lyxitol	1.15%	1.9	1.0
Xylitol	0.16%	1.2	0.97
Pseudouridine	1.77%	1.9	1.0
**Time 4**	**MDA**	**VIP score**	**ROC AUC**
Tryptophan	0.44%	1.5	0.97
Taurine	0.46%	1.4	0.93
Threonic acid	0.61%	1.4	0.97
Tyrosine	1.31%	1.8	1.0
Lyxitol	1.47%	1.6	1.0
Xylitol	0.52%	1.5	0.93
Pseudouridine	0.31%	1.2	0.93
**Time 5**	**MDA**	**VIP score**	**ROC AUC**
Tryptophan	-0.07%	1.2	0.9
Taurine	0.40%	1.5	0.93
Threonic acid	-0.23%	1.3	0.87
Tyrosine	2.85%	2.1	1.0
Lyxitol	0.33%	1.5	0.9
Xylitol	0.08%	1.4	0.87
Pseudouridine	0.58%	1.6	0.9

**Table 3:** Binary classification for the 7 metabolite model. These values were derived from multivariate exploratory ROC analysis with PLS-DA or RF as the final classification methods. The 7 metabolite model is composed of tyrosine, tryptophan, threonic acid, pseudouridine, taurine, xylitol, and lyxitol. Sens, sensitivity; Spec, specificity. When both training and testing time points are listed, the model is trained using data from training time point and used to predict the testing time point classifier (i.e treated vs control).

Classification Method	Training Time Point	Testing Time Point	True pos.	False neg.	True neg.	False pos.	Sens.	Spec.
PLS-DA	1	NA	3	2	3	3	60%	50%
PLS-DA	2	NA	5	0	6	0	100%	100%
PLS-DA	3	NA	5	0	6	0	100%	100%
PLS-DA	4	NA	5	0	6	0	100%	100%
PLS-DA	5	NA	5	0	5	1	100%	83.33%
RF	1	NA	2	3	2	4	40%	66.67%
RF	2	NA	4	1	6	0	80%	100%
RF	3	NA	5	0	5	1	100%	83.33%
RF	4	NA	5	0	6	0	100%	100%
RF	5	NA	5	0	5	1	100%	83.33%
PLS-DA	2	2	4	0	4	0	100%	100%
PLS-DA	4	3	0	4	3	1	0%	75%
PLS-DA	5	4	4	0	2	2	100%	50%
RF	2	2	3	1	3	1	75%	75%
RF	4	3	0	4	2	2	0%	50%
RF	5	4	3	1	3	1	75%	75%

### 3.5 Testing metabolome data set

All animals were clinically healthy according to physical examinations, cellular blood counts and blood chemistry panels before the administration of the treatments. Once treatments were started, cats in the control group remained healthy for the duration of the study. One cat in the control group vomited once on day 4 after the first drug administration. In the meloxicam treated group, the cats’ body-weights and condition scores were relatively stable.

#### 3.5.1 Kidney histopathology for testing data set

Following 17 days of consecutive subcutaneous meloxicam treatment every 24h, renal histologic lesions within the treatment group (n = 4) included multifocal cortical tubular dilation, proximal tubular epithelial degeneration and necrosis, degeneration and swelling of medullary tubular epithelial cells, heterogeneous cortical peri-degenerate tubular inflammatory infiltrate composed of peri-degenerate tubular aggregates of lymphocytes, plasma cells macrophages with fewer neutrophils and rare eosinophil. In contrast, cats within the saline control group (n = 4) had no to scattered tubular degeneration and minimal interstitial inflammatory infiltrate. These findings are consistent with acute kidney injury predominantly focused on the renal tubules. Representative photomicrographs, histologic scores, and aggregates scores for each cat are available in the supplementary data (S4 and S5 Figs, S4 Table).

#### 3.5.2 Testing data set creatinine concentrations and USG

The serum creatinine and USG values followed a similar clinical progression as observed in the training data set. Serum creatinine concentrations rose starting on day 7 and stayed elevated for the remainder of the study (17 days) consistent with decreased glomerular filtration rate (S6 Fig). Similarly, USG values did not drop below the cut-off of 1.035 until day 11 within the meloxicam treated group, consistent with renal tubular concentrating ability (S7 Fig).

#### 3.5.3 Validation of putative biomarkers using testing metabolome data set

The seven metabolite model was applied to the testing data set obtained from a separate meloxicam treatment experiment to confirm the value of these biomarkers in identifying cats treated with meloxicam.

A model using multivariate ROC analysis with either PLS-DA or RF as the classifier was fitted using the 7 putative biomarkers in the training data set and subsequently employed to predict the classification of unlabeled treated and control cats in the testing data set. The sensitivity and specificity for the seven metabolite complete model when applied to the test data set at each time point is reported in Table 3.

## 4. Discussion

In this study, we utilized an untargeted metabolomics approach to identify urine LMWs that could be further studied as biomarker candidates for monitoring during the administration of NSAIDs in cats. The subcutaneous administration of meloxicam rapidly and consistently altered the urine metabolome of cats with marked changes as early as 2 days (training data set time point 2) following the administration of meloxicam.

One of the earliest and strongest metabolic changes induced by meloxicam was a decrease in the intensity of most of the urinary metabolites (S5 Table). The exact mechanism for a decrease in the urine metabolites is unclear. Humans with chronic kidney disease also had decrease of several metabolites in urine [32]. However, our findings contrast with some earlier studies done in rodents reporting increased amino acid excretion upon dosing with cisplatin, aminoglycosides, and other nephrotoxins [33], [34], [35], [36], [37]. Under nephrotoxic conditions, amino acid excretion increases because of impaired reabsorption by the renal tubules, increased cellular turnover, or increased permeability of the glomerular membranes [38]. In our study, there was a decrease in the USG that was evident in most of the training data set cats after 13 days of treatment. A decrease in the USG is likely a result of alteration of the urine concentration capacity of kidneys. Moreover a decrease in the secretion or increase of reabsorption of metabolites could also explain a decrease in the intensity of some urinary metabolites. Interspecies, and experimental model differences and meloxicam-specific effects may be important factors dictating the studies dissimilarities. Another study suggests that the NSAID indomethacin increases solute reabsorption at the medullary segment of the thick ascending limb of the loop of Henle [39]. Whether meloxicam has similar effects as indomethacin in the kidney remains to be determined but it could also explain, at least partially, a lower urinary intensity of some metabolites.

Piroxicam (a drug that belongs to the same family of drugs as meloxicam) and other NSAIDs have been reported to inhibit organic anion transporters (hOATs) and human organic cation transporters (hOCTs). The exact mechanism of solutes’ renal excretion in cats is unknown but it is possible that at least some of the metabolites reported in this study are excreted by transport-mediated mechanisms which could have been altered by meloxicam [40].

A group of 7 LMWs, including taurine, tryptophan, tyrosine, lyxitol, pseudouridine, xylitol and threoinic acid were consistently able to discern meloxicam-treated from saline-treated cats in the training data set. The alterations in peak intensity of these metabolites in urine may be the result of kidney damage caused by the repeated administration of meloxicam. Urine samples can be considered a liquid biopsy of the kidneys [10], thus some of the changes in the urine metabolome might reflect effects of meloxicam on this organ. Interesting, within the training data set all these LMWs changed in urine earlier (at least 4 days after the first treatment) than creatinine (~10 days after the first treatment) in plasma, suggesting that these molecules could be used as urinary biomarkers for early detection of meloxicam-induced kidney damage. However, the metabolite changes in urine observed in this study cannot be solely associated with kidney damage as NSAIDs have effects in a myriad of body cells and organs which could also be reflected in the urine metabolome [4], [41], [42].

Notably, taurine, tryptophan, tyrosine, and pseudouridine were identified as potential biomarker candidates of kidney disease in other species [43], [44], [45], [46], [47], [48]. Several of the putative biomarkers proposed here have previously been shown to play a biologically significant role both within normal renal homeostasis and disease states. For example, taurine, a β-amino acid, is involved in regulation of renal blood flow [49, 50], apoptosis of renal tubular epithelial cells [51], [52] and protection against renal injury [53], [54]. Tyrosine, a non-essential amino acid, is produced through metabolism of phenylalanine by renal tubular epithelial cells. As renal function decreases following injury, there is decreased production of tyrosine.

ROC analysis results suggest that a cats’ phenotype can be discriminated based on seven metabolites identified in urine metabolome across multiple time points, with an average sensitivity of 95–100% and specificity of 91.7–95.8% in the training data set, depending on the classification algorithm utilized. Even though it is necessary to assess the robustness of the selected biomarkers through validation of the findings using independent data sets [55, 56], biological validation of putative biomarkers is rarely found in scientific biomarker discovery publications but necessary for increasing the value of the experimental findings. Distinctively, this study tested prospectively sensitivity and specificity of the 7 metabolites (from the training data set) on a separate metabolome data set (testing dataset) generated in an independent feline study. When the 7 metabolites are applied to a separate test data set this resulted in an average of 66–75%% sensitivity and 50–66% specificity across multiple time points. If testing time point 3 is excluded from the analysis, this increased the sensitivity and specificity to 75–100% and 75% respectively. These results using an independent data set support the relevance of the seven metabolite biomarkers for dichotomizing meloxicam-treated from saline-treated cats. When interpreting the calculated binary classification values it should be cautioned that these values are limited by the small sample size of this study. Additional studies in a larger population is required to confirm the diagnostic potential of the metabolites identified in this study.

This study discovered LMWs that are altered by the repeated administration of meloxicam and could be used to detect or predict early adverse effects in cats. However, the seven metabolites identified in this study should not be considered biomarkers yet because this study has several limitations. First, this pilot exploratory study has been performed in a relatively small and highly homogenous population of intact female domestic cats maintained under experimentally controlled conditions. Many extrinsic factors have been shown to affect the urinary metabolome including the gastrointestinal microbiota [57], [58], obesity/diet [59], [60], age [61], exercise [62], diurnal variation [63], gender [61], and environmental chemicals [64]. The elegance of the study reported here lies in the fact that we tested the metabolite selection using a separate data set generated during an independent study that included cats from a different breeder source, hosting conditions, and sampling times which increases the validity of this study’s findings. However, the data presented in this study should be considered for hypothesis generation. The validity of the metabolites found in this research should be assessed in studies including both genders, different ages, diets and other environmental conditions. Even though the sample sizes were enough to detect differences in the urine metabolome a larger study will increase the robustness and external validity of the present study’s findings. It is important to remark that this study provides, untargeted and nonbiased information regarding urinary metabolites, as the hypothesis is not based on pre-selection of potential candidates to be investigated. Nevertheless, currently, there is no single-instrument platform that can cover all urine metabolites (>3000) [65] therefore the LMWs evaluated in this study are likely to be a fraction of the actual urine metabolome because this study was limited to one analytical platform [18]. It is expected that expanding the metabolome analytical platforms will help to discover other metabolites that could serve as biomarkers for monitoring cats treated with NSAIDs.

## 5. Conclusions

This is the first study reporting changes in the urinary metabolome of cats with meloxicam-induced kidney disease. Using a machine leaning technique, this study identified a novel panel of 7 metabolites that may help to identify early non-symptomatic effects of the repeated administration of meloxicam. This panel of metabolites could be useful for drug development and clinical trials involving NSAIDs or monitoring clinical patients. However, the clinical value and robustness of this panel of metabolites need to be assessed in future experiments with a larger target population of animals.

## Supporting information

S1 Fig Alterations in serum creatinine concentrations for training data set cats treated with saline (n = 6) or meloxicam (n = 5) at 0.3 mg/kg every 24 hr for up to 47 days. The dashed line indicates a USG of 1.035. Urine samples were collected on days -3, 0, 4, 9, 13, 17, 23, 26, 31, 34, 40 and 47.
(DOCX)

S2 Fig Urine specific gravity values obtained by refractometry for the training data set. Cats were treated with saline (n = 6) or meloxicam (n = 5) at 0.3 mg/kg every 24 hr for up to 47 days. The dashed line indicates a USG of 1.035. Urine samples were collected on days -3, 0, 4, 9, 13, 17, 23, 26, 31, 34, 40 and 47.
(DOCX)

S3 Fig Supervised PLS-DA score plots performed on training data set urine metabolites obtained at sampling time points 1–5 from saline treated control cats (n = 6) and meloxicam treated cats (n = 5) at 0.3 mg/kg every 24 hr for up to 17 days, represented by triangles and crosses, respectively. Each data point represents a urine sample from an individual cat. Axis values are normalized principal component scores. Shaded backgrounds define the 95% confidence interval for each group. The sampling time point is denoted in the upper left of each plot.
(DOCX)

S4 Fig Photomicrographs of representative sections from either control cats treated with saline (n = 4) or meloxicam (n = 4) at 0.3 mg/kg every 24 hrs for 17 days. A) Kidney, cortical, control, testing data set. Normal cortical tubular components including proximal convoluted tubule with intracytoplasmic lipid vacuoles, thick ascending limb of the nephron and a glomerulus, hematoxylin and eosin (H&E, 200x). B) Kidney, cortical, meloxicam treated, testing data set. Renal tubular dilation, tubular epithelial cell degeneration and interstitial mixed inflammation (H&E, 200x). C) Kidney, cortical, meloxicam treated, testing data set. Tubular degeneration characterized by tubular epithelial cells with granular, faintly eosinophilic and vacuolated cytoplasm with renal tubular epithelial necrosis (H&E, 400x). D) Kidney, medullary, meloxicam treated. Nodular accumulation of lymphocytes, neutrophils and macrophages admixed with necrotic cellular debris within the medullary interstitium (H&E, 400x). E) Kidney, cortical, control, testing data set. Diffusely intact and histologically unremarkable renal tubular basement membranes (PAMS, 200x). F) Kidney, cortical, meloxicam treated, testing data set. Multifocal fraying and loss of basement membrane integrity which correlates to regions of interstitial inflammation and tubular necrosis in the H&E replicate sections (PAMS, 200x).
(DOCX)

S5 Fig Box and whisker plot for the aggregate semi-quantitative renal histologic scores for the testing data set obtained from blinded examination of at least one kidney from each control cat treated with saline (n = 4) or meloxicam (n = 4) at 0.3 mg/kg every 24 hr for 17 days. The bold center line represents the median score, the boxes above and below the median line are the first and third quartile. The whiskers represent the maximum and minimum scores for each group. Utilizing the non-parametric Mann-Whiney test, there is a significant difference in renal histologic scores between the meloxicam treated group (M) and the saline treated control cats (C) (*p* = 0.006766).
(DOCX)

S6 Fig Alterations in serum creatinine concentrations for testing data set cats treated with saline (n = 4) or meloxicam (n = 4) at 0.3 mg/kg every 24 hr for 17 days. Creatinine values are displayed in percent change from baseline samples. Serum samples were collected on days -1, 1, 11, 14 and 17.
(DOCX)

S7 Fig Urine specific gravity values obtained by refractometry for the testing data. Cats were treated with saline (n = 4) or meloxicam (n = 4) at 0.3 mg/kg every 24 hr for 17 days. The dashed line indicates a USG of 1.035. Urine samples were collected on days -1, 2, 11, 14 and 17.
(DOCX)

S1 Table List of variable importance in projection (VIP) scores calculated from partial least squares discriminant analysis (PLS-DA) performed on training data set urine metabolites. The list of identified metabolites is derived from urine collected from control cats treated with saline (n = 6) or meloxicam (n = 5) at 0.3 mg/kg every 24 hr for up to 17 days (time point 5). Metabolites with VIP scores > 1 were considered for inclusion in the model. Blank values indicate the metabolite was not detected at that time point or was removed during the data filtering step.
(DOCX)

S2 Table List of mean decrease in accuracy (MDA) scores calculated using the machine learning algorithm, random forest classification methods performed on training data set urine metabolites identified in this study. The list of identified metabolites is derived from urine collected from control cats treated with saline (n = 6) or meloxicam (n = 5) at 0.3 mg/kg every 24 hr for up to 17 days (time point 5). Metabolites with MDA scores > 0.0004 were considered for inclusion in the model. Blank values indicate the metabolite was not detected at that time point or was removed during the data filtering step.
(DOCX)

S3 Table List of area under the curve (AUC) values calculated using receiver operator curve analysis methods performed on training data set urine metabolites identified in this study. The list of identified metabolites is derived from urine collected from control cats treated with saline (n = 6) or meloxicam (n = 5) at 0.3 mg/kg every 24 hr for up to 17 days (time point 5). Metabolites with AUC scores > 0.85 were considered for inclusion in the model. Metabolites with AUC scores > 0.85 at time point 1 were excluded from the model. Blank values indicate the metabolite was not detected at that time point or was removed during the data filtering step.
(DOCX)

S4 Table Raw semi-quantitative renal histologic scores for the testing data set obtained from blinded examination of at least one kidney from each control cat treated with saline (n = 4) or meloxicam (n = 4) at 0.3 mg/kg every 24 hrs for 17 days. Tubule damage and inflammation were assessed on hematoxylin and eosin (H&E) stained sections and basement membrane integrity were assessed on periodic acid-Schiff methenamine silver (PAMS) stained sections. Cortical and corticomedullary tubules were assessed for degeneration, necrosis, regeneration, dilation, attenuation and hypertrophy. Any subtype of inflammatory cell within the interstitium (i.e. neutrophil, lymphocyte, macrophage, eosinophil, etc.) were counted when determining extent of inflammation. The maximum score per category is 4 with a maximum possible score of 16.
(DOCX)

S5 Table List of average, maximum, and minimum raw intensity values (m/z) obtained from GC-MS assay for each time point. The list of identified metabolites is derived from training data set urine collected from control cats treated with saline (n = 6) or meloxicam (n = 5) at 0.3 mg/kg every 24 hrs for up to 17 days (time point 5). Values highlighted in red indicate an increase in average intensity (> 1.2 fold) and values highlighted in yellow represent a decrease in average intensity (< 0.8) when comparing the meloxicam treated group vs. the saline treated control group.
(DOCX)
