# Supplementary material for: Urinary chemical fingerprint left behind by repeated NSAID administration: Discovery of putative biomarkers using artificial intelligence
Source: PLoS One. 2020 Feb 13;15(2):e0228989. doi: 10.1371/journal.pone.0228989 (PMC7018043; doi:10.1371/journal.pone.0228989)
Supplement: S2 Fig — Cats were treated with saline (n = 6) or meloxicam (n = 5) at 0.3 mg/kg every 24 hr for up to 47 days. The dashed line indicates a USG of 1.035. Urine samples were collected on days -3, 0, 4, 9, 13, 17, 23, 26, 31, 34, 40 and 47. (DOCX) [file pone.0228989.s002.docx]

**Supplemental Figure S2:** Urine specific gravity values obtained by refractometry for the training data set**.** Cats were treated with saline (n=6) or meloxicam (n=5) at 0.3 mg/kg every 24 hr for up to 47 days. The dashed line indicates a USG of 1.035. Urine samples were collected on days -3, 0, 4, 9, 13, 17, 23, 26, 31, 34, 40 and 47.
