# Supplementary material for: Urinary chemical fingerprint left behind by repeated NSAID administration: Discovery of putative biomarkers using artificial intelligence
Source: PLoS One. 2020 Feb 13;15(2):e0228989. doi: 10.1371/journal.pone.0228989 (PMC7018043; doi:10.1371/journal.pone.0228989)
Supplement: S3 Fig — Each data point represents a urine sample from an individual cat. Axis values are normalized principal component scores. Shaded backgrounds define the 95% confidence interval for each group. The sampling time point is denoted in the upper left of each plot. (DOCX) [file pone.0228989.s003.docx]

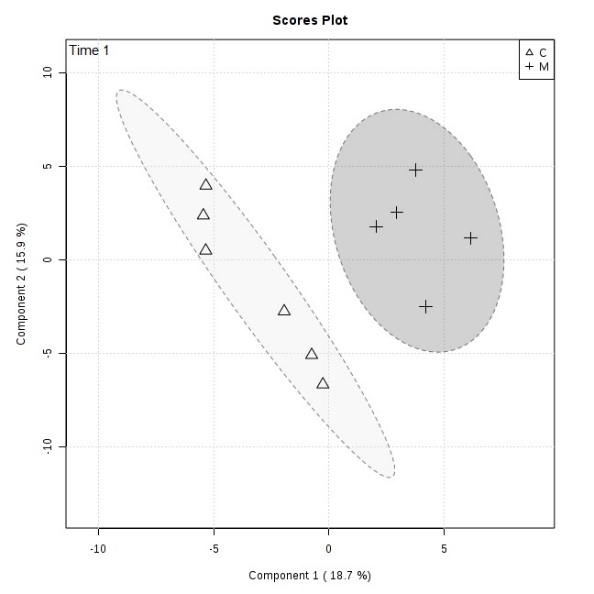

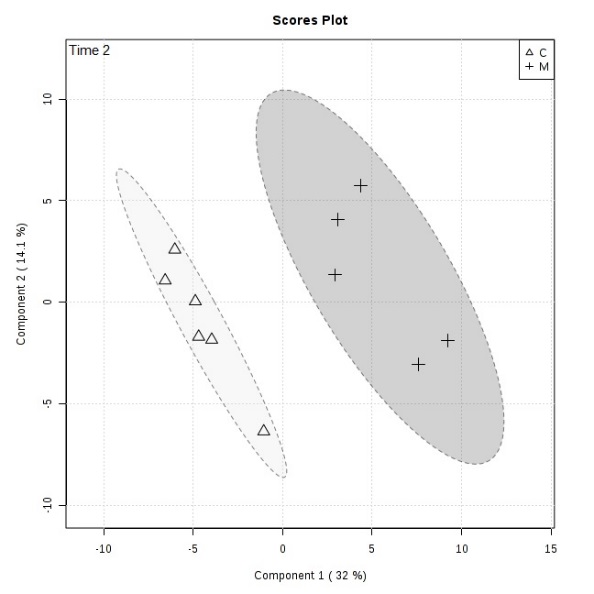


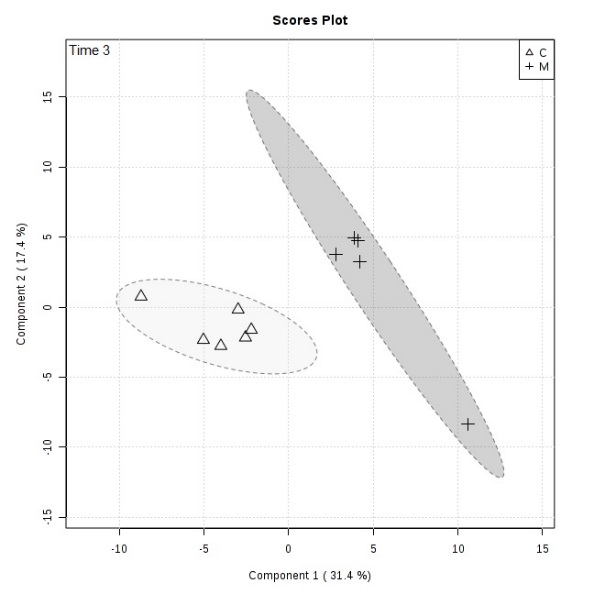

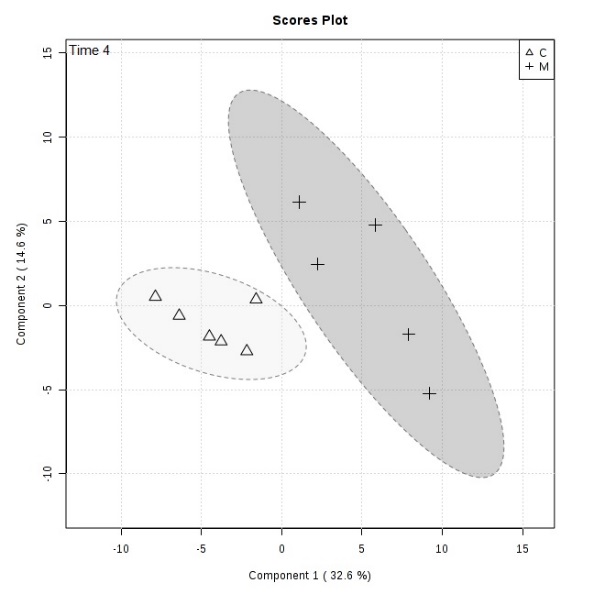

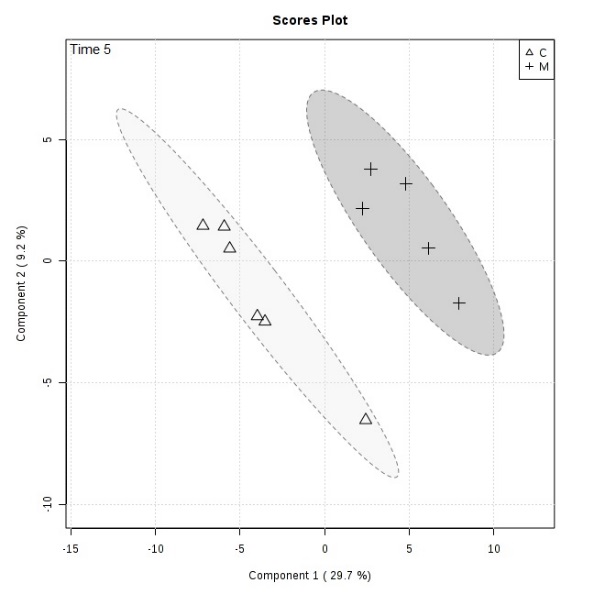


**Supplemental Figure S3:** Supervised PLS-DA score plots performed on training data set urine metabolites obtained at sampling time points 1-5 from saline treated control cats (n=6) and meloxicam treated cats (n=5) at 0.3 mg/kg every 24 hr for up to 17 days, represented by triangles and crosses, respectively. Each data point represents a urine sample from an individual cat. Axis values are normalized principal component scores. Shaded backgrounds define the 95% confidence interval for each group. The sampling time point is denoted in the upper left of each plot.
