# Supplementary material for: Urinary chemical fingerprint left behind by repeated NSAID administration: Discovery of putative biomarkers using artificial intelligence
Source: PLoS One. 2020 Feb 13;15(2):e0228989. doi: 10.1371/journal.pone.0228989 (PMC7018043; doi:10.1371/journal.pone.0228989)
Supplement: S4 Fig — A) Kidney, cortical, control, testing data set. Normal cortical tubular components including proximal convoluted tubule with intracytoplasmic lipid vacuoles, thick ascending limb of the nephron and a glomerulus, hematoxylin and eosin (H&E, 200x). B) Kidney, cortical, meloxicam treated, testing data set. Renal tubular dilation, tubular epithelial cell degeneration and interstitial mixed inflammation (H&E, 200x). C) Kidney, cortical, meloxicam treated, testing data set. Tubular degeneration characterized by tubular epithelial cells with granular, faintly eosinophilic and vacuolated cytoplasm with renal tubular epithelial necrosis (H&E, 400x). D) Kidney, medullary, meloxicam treated. Nodular accumulation of lymphocytes, neutrophils and macrophages admixed with necrotic cellular debris within the medullary interstitium (H&E, 400x). E) Kidney, cortical, control, testing data set. Diffusely intact and histologically unremarkable renal tubular basement membranes (PAMS, 200x). F) Kidney, cortical, meloxicam treated, testing data set. Multifocal fraying and loss of basement membrane integrity which correlates to regions of interstitial inflammation and tubular necrosis in the H&E replicate sections (PAMS, 200x). (DOCX) [file pone.0228989.s004.docx]

A

B

C

D

E

F

**Supplemental Figure S4:** Photomicrographs of representative sections from either control cats treated with saline (n=4) or meloxicam (n=4) at 0.3 mg/kg every 24 hrs for 17 days. A) Kidney, cortical, control, testing data set. Normal cortical tubular components including proximal convoluted tubule with intracytoplasmic lipid vacuoles, thick ascending limb of the nephron and a glomerulus, hematoxylin and eosin (H&E, 200x). B) Kidney, cortical, meloxicam treated, testing data set. Renal tubular dilation, tubular epithelial cell degeneration and interstitial mixed inflammation (H&E, 200x). C) Kidney, cortical, meloxicam treated, testing data set. Tubular degeneration characterized by tubular epithelial cells with granular, faintly eosinophilic and vacuolated cytoplasm with renal tubular epithelial necrosis (H&E, 400x). D) Kidney, medullary, meloxicam treated. Nodular accumulation of lymphocytes, neutrophils and macrophages admixed with necrotic cellular debris within the medullary interstitium (H&E, 400x). E) Kidney, cortical, control, testing data set. Diffusely intact and histologically unremarkable renal tubular basement membranes (PAMS, 200x). F) Kidney, cortical, meloxicam treated, testing data set. Multifocal fraying and loss of basement membrane integrity which correlates to regions of interstitial inflammation and tubular necrosis in the H&E replicate sections (PAMS, 200x).
