# Supplementary material for: Urinary chemical fingerprint left behind by repeated NSAID administration: Discovery of putative biomarkers using artificial intelligence
Source: PLoS One. 2020 Feb 13;15(2):e0228989. doi: 10.1371/journal.pone.0228989 (PMC7018043; doi:10.1371/journal.pone.0228989)
Supplement: S5 Fig — The bold center line represents the median score, the boxes above and below the median line are the first and third quartile. The whiskers represent the maximum and minimum scores for each group. Utilizing the non-parametric Mann-Whiney test, there is a significant difference in renal histologic scores between the meloxicam treated group (M) and the saline treated control cats (C) (p = 0.006766). (DOCX) [file pone.0228989.s005.docx]

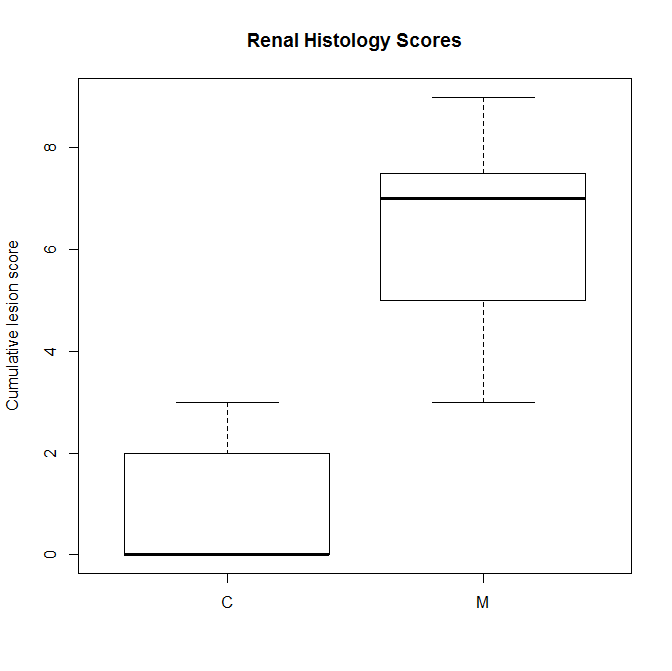


**Supplemental Figure S5:** Box and whisker plot for the aggregate semi-quantitative renal histologic scores for the testing data set obtained from blinded examination of at least one kidney from each control cat treated with saline (n=4) or meloxicam (n=4) at 0.3 mg/kg every 24 hr for 17 days. The bold center line represents the median score, the boxes above and below the median line are the first and third quartile. The whiskers represent the maximum and minimum scores for each group. Utilizing the non-parametric Mann-Whiney test, there is a significant difference in renal histologic scores between the meloxicam treated group (M) and the saline treated control cats (C) (*p*= 0.006766).
