# Supplementary material for: Urinary chemical fingerprint left behind by repeated NSAID administration: Discovery of putative biomarkers using artificial intelligence
Source: PLoS One. 2020 Feb 13;15(2):e0228989. doi: 10.1371/journal.pone.0228989 (PMC7018043; doi:10.1371/journal.pone.0228989)
Supplement: S6 Fig — Creatinine values are displayed in percent change from baseline samples. Serum samples were collected on days -1, 1, 11, 14 and 17. (DOCX) [file pone.0228989.s006.docx]

**Supplemental Figure S6:** Alterations in serum creatinine concentrations for testing data set cats treated with saline (n=4) or meloxicam (n=4) at 0.3 mg/kg every 24 hr for 17 days. Creatinine values are displayed in percent change from baseline samples. Serum samples were collected on days -1, 1, 11, 14 and 17.
