# Supplementary material for: Urinary chemical fingerprint left behind by repeated NSAID administration: Discovery of putative biomarkers using artificial intelligence
Source: PLoS One. 2020 Feb 13;15(2):e0228989. doi: 10.1371/journal.pone.0228989 (PMC7018043; doi:10.1371/journal.pone.0228989)
Supplement: S7 Fig — Cats were treated with saline (n = 4) or meloxicam (n = 4) at 0.3 mg/kg every 24 hr for 17 days. The dashed line indicates a USG of 1.035. Urine samples were collected on days -1, 2, 11, 14 and 17. (DOCX) [file pone.0228989.s007.docx]

**Supplemental Figure S7:** Urine specific gravity values obtained by refractometry for the testing data**.** Cats were treated with saline (n=4) or meloxicam (n=4) at 0.3 mg/kg every 24 hr for 17 days. The dashed line indicates a USG of 1.035. Urine samples were collected on days -1, 2, 11, 14 and 17.
