# Supplementary material for: Urinary chemical fingerprint left behind by repeated NSAID administration: Discovery of putative biomarkers using artificial intelligence
Source: PLoS One. 2020 Feb 13;15(2):e0228989. doi: 10.1371/journal.pone.0228989 (PMC7018043; doi:10.1371/journal.pone.0228989)
Supplement: S1 Table — The list of identified metabolites is derived from urine collected from control cats treated with saline (n = 6) or meloxicam (n = 5) at 0.3 mg/kg every 24 hr for up to 17 days (time point 5). Metabolites with VIP scores > 1 were considered for inclusion in the model. Blank values indicate the metabolite was not detected at that time point or was removed during the data filtering step. (DOCX) [file pone.0228989.s008.docx]

**Supplemental Table S1:** List of variable importance in projection (VIP) scores calculated from partial least squares discriminant analysis (PLS-DA) performed on training data set urine metabolites. The list of identified metabolites is derived from urine collected from control cats treated with saline (n=6) or meloxicam (n=5) at 0.3 mg/kg every 24 hr for up to 17 days (time point 5). Metabolites with VIP scores > 1 were considered for inclusion in the model. Blank values indicate the metabolite was not detected at that time point or was removed during the data filtering step.

| **Metabolite** | **Time 1** | **Time 2** | **Time 3** | **Time 4** | **Time 5** |
| --- | --- | --- | --- | --- | --- |
| 3,4-dihydroxycinnamic acid | 0.10081 | 0.66311 | 1.1878 | 1.3929 | 0.98874 |
| 3,4-dihydroxyhydrocinnamic acid NIST | 1.0913 | 0.14642 | 0.70988 | 1.1037 | 0.000106 |
| 3,4-dihydroxyphenylacetic acid | 1.0995 | 0.28576 | 0.83852 | 0.012697 | 0.2295 |
| 3-3-hydroxyphenylpropionic acid | 1.4789 | 0.50116 | 1.1543 | 0.56709 | 0.51229 |
| 3-4-hydroxyphenylpropionic acid | 0.049808 | 0.22971 | 1.3141 | 0.72123 | 1.276 |
| 3-aminoisobutyric acid | 1.0381 | 0.49128 | 0.67465 | 0.1223 | 0.85061 |
| 3-hydroxy-3-methylglutaric acid | 1.7926 | 1.2105 | 1.0904 | 0.49076 | 1.474 |
| 3-phosphoglycerate | 0.55737 | 0.38363 | 0.87078 | 0.92587 | 0.86739 |
| 4-hydroxybenzoate | 0.50548 | 0.91302 | 0.22036 | 0.40809 | 0.39574 |
| 4-hydroxybutyric acid | 1.7303 | 1.407 | 0.768 | 1.0666 | 1.2705 |
| 4-hydroxyhippuric acid NIST | 1.0698 | 0.90892 | 1.0151 | 0.13612 | 1.0118 |
| 4-hydroxyphenylacetic acid | 1.739 | 1.0422 | 0.57086 | 0.50781 | 1.2301 |
| 4-hydroxyphenylacetic acid |  |  | 0.046933 | 0.31722 | 0.50268 |
| 5-hydroxy-3-indoleacetic acid | 0.5527 | 1.0671 | 1.2833 | 1.5632 | 1.064 |
| aconitic acid | 1.1772 | 0.33893 | 0.93273 | 1.1854 | 0.60573 |
| adenosine | 0.15186 | 1.1556 | 1.6842 | 1.5288 | 1.3748 |
| alanine | 0.85965 | 0.9443 | 0.72398 | 0.37229 | 0.29855 |
| allantoic acid | 0.16202 | 0.79635 | 0.70195 | 0.27931 | 1.2371 |
| alpha-ketoglutarate | 2.1002 | 1.3032 | 0.74642 | 0.71975 | 1.6659 |
| benzoic acid | 0.33789 | 0.82722 | 0.58637 | 1.3436 | 0.46577 |
| benzylalcohol | 0.53311 |  | 1.018 | 0.75776 | 0.91943 |
| beta-alanine | 1.1701 | 0.29132 | 0.046884 | 0.89202 | 0.75641 |
| beta-gentiobiose | 0.33175 | 1.2829 | 0.63621 | 1.3408 | 0.7239 |
| catechol | 1.2168 | 0.5561 | 0.47244 | 0.59661 | 0.38607 |
| citramalic acid | 0.64247 | 0.9056 | 0.59578 | 0.045089 | 1.574 |
| citric acid | 2.1358 | 1.5829 | 1.199 | 1.0894 | 1.9536 |
| citrulline | 2.0199 | 0.9552 | 1.3567 | 1.3086 | 1.5866 |
| conduritol-beta-expoxide | 0.21137 | 0.98806 | 0.34952 | 0.7175 | 0.31978 |
| creatinine | 0.59433 | 1.0683 | 1.6665 | 1.0252 | 1.0142 |
| deoxypentitol | 0.6538 | 1.3318 | 1.3545 | 1.0832 | 1.4135 |
| erythritol | 0.29759 | 0.75879 | 0.70243 | 0.94897 | 0.49345 |
| ferulic acid | 0.91534 | 0.78965 | 0.94439 | 1.5574 | 0.70339 |
| fructose | 0.97916 | 1.4329 | 1.1658 | 1.3201 | 0.42866 |
| fucose | 1.1877 | 1.1391 | 1.3406 | 1.0825 | 1.3555 |
| galactinol | 0.93268 | 0.77503 | 0.75095 | 0.69548 | 1.053 |
| galactonic acid | 1.2934 | 0.82569 | 0.33304 | 1.5999 | 1.872 |
| gluconic acid | 1.0654 | 1.2157 | 1.1817 | 0.099358 | 1.0036 |
| glyceric acid | 0.23746 | 0.40616 | 0.38199 | 0.94771 | 1.2636 |
| glycerol | 1.1756 | 0.69079 | 0.009548 | 1.291 | 0.32493 |
| glycerol-3-galactoside | 1.3805 | 0.97717 | 1.6293 | 1.1227 | 1.8336 |
| glycine | 0.66524 | 1.0214 | 0.69568 | 0.89271 | 0.10705 |
| glycocyamine | 0.065821 | 1.6117 | 1.7812 | 0.90291 | 1.0946 |
| glycolic acid | 1.2032 | 0.63579 | 0.39406 | 0.84564 | 0.81405 |
| hexadecane | 0.33397 | 0.4787 | 0.8112 | 1.1307 | 1.2042 |
| hexitol | 0.17609 | 1.1099 | 0.59898 | 0.49961 | 0.10429 |
| hexuronic acid | 0.02333 | 1.0382 | 0.1383 | 0.025369 | 0.61184 |
| hippuric acid | 1.249 | 1.0802 | 0.94101 | 1.2036 | 0.81573 |
| hydroxylamine | 0.67069 | 0.92692 | 0.56253 | 0.95696 | 0.35055 |
| indole-3-acetate | 0.20416 | 0.54822 | 0.84265 | 1.2284 | 0.38289 |
| indoxyl sulfate | 0.63838 | 0.71672 | 0.24615 | 0.046268 | 0.25191 |
| inosine | 0.51833 | 0.14377 | 0.40483 |  |  |
| isocitric acid | 1.376 | 0.1774 | 0.77312 | 0.98093 | 0.24243 |
| isohexonic acid | 0.33438 | 0.64553 | 0.024505 | 0.47319 | 0.99911 |
| isomaltose | 0.4704 | 0.66602 | 0.20559 | 0.12118 | 1.0387 |
| isoribose | 0.3363 | 1.1251 | 1.3474 | 1.3694 | 1.2398 |
| isothreonic acid | 0.73749 | 0.9985 | 0.99172 | 0.11574 | 0.75105 |
| kynurenic acid | 0.38318 | 1.0642 | 1.5786 |  | 1.8202 |
| lactic acid | 0.70228 | 0.71029 | 1.0889 | 1.5552 | 0.90196 |
| lysine | 0.27783 | 1.6038 | 1.8479 | 1.0076 | 1.1862 |
| lyxitol | 0.51433 | 1.353 | 1.8889 | 1.6445 | 1.5326 |
| lyxose | 0.83142 | 0.30731 | 0.83052 | 0.19351 | 0.20741 |
| malic acid | 1.7674 | 1.068 | 0.26111 | 1.0671 | 1.5594 |
| mannose | 2.0712 | 1.0549 | 0.81502 | 0.17381 | 0.32425 |
| mucic acid | 1.7124 | 0.87121 | 0.022897 | 1.2172 | 1.4266 |
| myo-inositol | 0.75697 | 0.92137 | 0.45216 | 0.23776 | 0.79585 |
| N-acetylaspartic acid | 0.15585 | 0.070617 | 0.33495 | 0.51411 | 0.47193 |
| n-acetyl-d-hexosamine | 0.12982 | 1.3505 | 1.2498 | 0.98774 | 0.77828 |
| N-acetylmannosamine | 0.60599 | 0.7589 | 1.1179 | 0.16611 | 0.6671 |
| ornithine | 1.6188 | 0.82715 | 0.66692 | 0.15691 | 0.85408 |
| oxalic acid | 1.7076 | 0.40644 | 0.89415 | 0.66788 | 1.4664 |
| oxoproline | 1.4253 | 1.5905 | 0.53064 | 1.2206 | 0.87116 |
| palmitic acid | 0.13164 | 1.1174 | 1.1097 | 1.1202 | 0.27608 |
| pelargonic acid | 0.11357 | 1.0916 | 0.26654 | 1.427 | 0.53244 |
| pentitol |  | 0.80502 |  | 0.23729 |  |
| phenaceturic acid | 0.16534 | 0.31824 | 0.23687 | 1.3021 | 0.77186 |
| phenol | 0.42557 | 1.3531 | 1.1694 | 1.0048 | 1.0529 |
| phosphate | 1.0571 | 0.10085 | 0.43864 | 0.55287 | 0.86625 |
| pimelic acid | 1.3747 | 1.6481 | 1.1505 | 1.2269 | 1.6576 |
| pinitol | 1.1771 | 1.3877 | 0.89631 | 1.3175 | 1.2589 |
| propane-1,3-diol NIST | 0.1744 | 0.56839 | 0.25012 | 0.74989 | 0.42694 |
| pseudo uridine | 0.79816 | 1.2656 | 1.918 | 1.1799 | 1.5645 |
| putrescine | 0.32085 | 1.7665 | 1.7042 | 1.0055 | 1.1064 |
| pyruvic acid | 0.44626 | 1.0263 | 0.4729 | 0.37762 | 0.31715 |
| quinic acid | 1.238 | 0.90067 | 0.319 |  | 0.066301 |
| raffinose | 0.97539 | 0.77814 | 0.47039 | 1.4284 | 0.25869 |
| ribitol | 0.018108 | 1.2131 | 1.316 | 0.90486 | 0.70822 |
| ribonic acid | 0.99039 | 0.90681 | 1.4427 | 0.88577 | 0.26968 |
| ribose | 0.76672 | 1.1638 | 1.3708 | 0.97074 | 0.41963 |
| saccharic acid | 0.37393 | 0.33417 | 0.76876 | 1.1741 | 0.91775 |
| serine | 0.4331 |  |  | 0.77351 | 1.4043 |
| sorbitol | 1.4226 | 0.75505 | 0.55926 | 0.25314 | 0.46917 |
| stearic acid | 0.021738 | 0.98905 | 0.96476 | 0.87072 | 0.41636 |
| succinic acid |  | 0.11975 | 1.1262 | 0.40451 | 0.54647 |
| sucrose | 0.15942 | 0.39467 | 0.050046 | 0.7379 | 0.22906 |
| sulfuric acid | 0.01923 | 0.86843 | 0.063908 | 0.28794 | 0.99056 |
| taurine | 1.1438 | 1.647 | 1.9861 | 1.4179 | 1.4628 |
| threitol | 1.1589 | 1.0202 | 1.0596 | 1.122 | 1.5578 |
| threonic acid | 0.95538 | 1.4979 | 1.3245 | 1.4003 | 1.3323 |
| trehalose | 0.81252 | 1.0544 | 0.8198 | 0.8269 | 0.26309 |
| tryptophan | 1.5029 | 1.6717 | 1.6521 | 1.5188 | 1.2074 |
| tyrosine | 1.5474 | 1.3543 | 1.5771 | 1.8355 | 2.0611 |
| tyrosol | 2.168 | 1.1823 | 0.50862 | 1.0222 | 0.099289 |
| urea | 0.034364 |  | 0.57702 | 1.3771 | 0.85302 |
| uric acid | 0.2774 | 1.2673 | 1.5305 | 1.4041 | 1.0692 |
| uridine | 1.0316 | 1.6122 | 0.99831 | 1.3892 | 0.62375 |
| valine | 0.9637 | 0.1984 | 1.0693 | 0.99175 | 0.052852 |
| vanillic acid | 1.3335 | 0.12071 | 0.82439 | 0.7067 | 0.83284 |
| xylitol | 0.69074 | 1.4223 | 1.1779 | 1.4592 | 1.3756 |
| xylonic acid | 1.0714 |  |  | 1.4852 |  |
| xylose | 1.2387 | 0.92338 | 1.3912 | 0.61978 | 0.22655 |
| xylulose NIST | 0.316 | 0.96401 | 1.1033 | 1.3218 | 0.95817 |
