# Supplementary material for: Urinary chemical fingerprint left behind by repeated NSAID administration: Discovery of putative biomarkers using artificial intelligence
Source: PLoS One. 2020 Feb 13;15(2):e0228989. doi: 10.1371/journal.pone.0228989 (PMC7018043; doi:10.1371/journal.pone.0228989)
Supplement: S3 Table — The list of identified metabolites is derived from urine collected from control cats treated with saline (n = 6) or meloxicam (n = 5) at 0.3 mg/kg every 24 hr for up to 17 days (time point 5). Metabolites with AUC scores > 0.85 were considered for inclusion in the model. Metabolites with AUC scores > 0.85 at time point 1 were excluded from the model. Blank values indicate the metabolite was not detected at that time point or was removed during the data filtering step. (DOCX) [file pone.0228989.s010.docx]

**Supplemental Table S3:** List of area under the curve (AUC) values calculated using receiver operator curve analysis methods performed on training data set urine metabolites identified in this study. The list of identified metabolites is derived from urine collected from control cats treated with saline (n=6) or meloxicam (n=5) at 0.3 mg/kg every 24 hr for up to 17 days (time point 5). Metabolites with AUC scores > 0.85 were considered for inclusion in the model. Metabolites with AUC scores > 0.85 at time point 1 were excluded from the model. Blank values indicate the metabolite was not detected at that time point or was removed during the data filtering step.

| **Metabolite** | **Time 1** | **Time 2** | **Time 3** | **Time 4** | **Time 5** |
| --- | --- | --- | --- | --- | --- |
| 3,4-dihydroxycinnamic acid | 0.5 | 0.66667 | 0.73333 | 0.86667 | 0.66667 |
| 3,4-dihydroxyhydrocinnamic acid NIST | 0.66667 | 0.56667 | 0.7 | 0.8 | 0.5 |
| 3,4-dihydroxyphenylacetic acid | 0.73333 | 0.63333 | 0.73333 | 0.66667 | 0.53333 |
| 3-3-hydroxyphenylpropionic acid | 0.83333 | 0.6 | 0.83333 | 0.53333 | 0.63333 |
| 3-4-hydroxyphenylpropionic acid | 0.63333 | 0.53333 | 0.86667 | 0.66667 | 0.83333 |
| 3-aminoisobutyric acid | 0.63333 | 0.5 | 0.66667 | 0.53333 | 0.66667 |
| 3-hydroxy-3-methylglutaric acid | 0.86667 | 0.8 | 0.76667 | 0.63333 | 0.86667 |
| 3-phosphoglycerate | 0.6 | 0.53333 | 0.76667 | 0.7 | 0.63333 |
| 4-hydroxybenzoate | 0.5 | 0.86667 | 0.56667 | 0.53333 | 0.56667 |
| 4-hydroxybutyric acid | 0.83333 | 0.93333 | 0.66667 | 0.73333 | 0.9 |
| 4-hydroxycinnamic acid | |  | 0.73333 | 0.6 | 0.73333 |
| 4-hydroxyhippuric acid NIST | 0.66667 | 0.8 | 0.66667 | 0.6 | 0.8 |
| 4-hydroxyphenylacetic acid | 0.83333 | 0.83333 | 0.56667 | 0.6 | 0.5 |
| 5-hydroxy-3-indoleacetic acid | 0.56667 | 0.8 | 0.83333 | 0.96667 | 0.76667 |
| aconitic acid | 0.76667 | 0.53333 | 0.73333 | 0.83333 | 0.63333 |
| adenosine | 0.53333 | 0.9 | 1 | 0.9 | 0.83333 |
| alanine | 0.7 | 0.73333 | 0.7 | 0.6 | 0.56667 |
| allantoic acid | 0.56667 | 0.56667 | 0.7 | 0.53333 | 0.8 |
| alpha-ketoglutarate | 0.93333 | 0.86667 | 0.8 | 0.66667 | 0.93333 |
| benzoic acid | 0.53333 | 0.73333 | 0.63333 | 0.83333 | 0.53333 |
| benzylalcohol | 0.53333 |  | 0.7 | 0.66667 | 0.73333 |
| beta-alanine | 0.73333 | 0.63333 | 0.6 | 0.66667 | 0.73333 |
| beta-gentiobiose | 0.5 | 0.86667 | 0.66667 | 0.9 | 0.7 |
| catechol | 0.76667 | 0.6 | 0.66667 | 0.66667 | 0.66667 |
| citramalic acid | 0.66667 | 0.63333 | 0.76667 | 0.63333 | 0.9 |
| citric acid | 0.93333 | 0.93333 | 0.86667 | 0.8 | 1 |
| citrulline | 0.93333 | 0.8 | 0.86667 | 0.83333 | 0.93333 |
| conduritol-beta-expoxide | 0.5 | 0.9 | 0.5 | 0.63333 | 0.66667 |
| creatinine | 0.66667 | 0.83333 | 0.96667 | 0.8 | 0.73333 |
| deoxypentitol | 0.56667 | 0.9 | 0.83333 | 0.73333 | 0.83333 |
| erythritol | 0.5 | 0.73333 | 0.66667 | 0.73333 | 0.56667 |
| ferulic acid | 0.63333 | 0.7 | 0.73333 | 0.9 | 0.7 |
| fructose | 0.66667 | 0.96667 | 0.8 | 0.93333 | 0.6 |
| fucose | 0.73333 | 0.83333 | 0.86667 | 0.8 | 0.86667 |
| galactinol | 0.56667 | 0.8 | 0.7 | 0.73333 | 0.83333 |
| galactonic acid | 0.73333 | 0.7 | 0.6 | 0.93333 | 0.93333 |
| gluconic acid | 0.66667 | 0.83333 | 0.83333 | 0.66667 | 0.73333 |
| glyceric acid | 0.63333 | 0.66667 | 0.6 | 0.76667 | 0.8 |
| glycerol | 0.73333 | 0.63333 | 0.53333 | 0.86667 | 0.63333 |
| glycerol-3-galactoside | 0.8 | 0.8 | 1 | 0.8 | 0.96667 |
| glycine | 0.6 | 0.83333 | 0.63333 | 0.73333 | 0.6 |
| glycocyamine | 0.53333 | 1 | 1 | 0.76667 | 0.8 |
| glycolic acid | 0.7 | 0.56667 | 0.5 | 0.7 | 0.8 |
| hexadecane | 0.6 | 0.56667 | 0.7 | 0.76667 | 0.83333 |
| hexitol | 0.56667 | 0.8 | 0.56667 | 0.53333 | 0.53333 |
| hexuronic acid | 0.63333 | 0.8 | 0.53333 | 0.63333 | 0.6 |
| hippuric acid | 0.76667 | 0.83333 | 0.8 | 0.9 | 0.73333 |
| hydroxylamine | 0.63333 | 0.8 | 0.56667 | 0.6 | 0.56667 |
| indole-3-acetate | 0.56667 | 0.8 | 0.73333 | 0.86667 | 0.63333 |
| indoxyl sulfate | 0.63333 | 0.73333 | 0.6 | 0.53333 | 0.53333 |
| inosine | 0.56667 | 0.6 | 0.53333 |  | 0.53333 |
| isocitric acid | 0.76667 | 0.53333 | 0.7 | 0.66667 | 0.5 |
| isohexonic acid | 0.6 | 0.66667 | 0.5 | 0.7 | 0.73333 |
| isomaltose | 0.56667 | 0.66667 | 0.5 | 0.53333 | 0.73333 |
| isoribose | 0.56667 | 0.76667 | 0.93333 | 0.9 | 0.83333 |
| isothreonic acid | 0.6 | 0.86667 | 0.73333 | 0.66667 | 0.63333 |
| kynurenic acid | 0.6 | 0.83333 | 0.9 |  | 1 |
| lactic acid | 0.6 | 0.73333 | 0.7 | 1 | 0.7 |
| lysine | 0.56667 | 0.96667 | 1 | 0.83333 | 0.9 |
| lyxitol | 0.53333 | 0.93333 | 1 | 1 | 0.9 |
| lyxose | 0.73333 | 0.6 | 0.7 | 0.73333 | 0.56667 |
| malic acid | 0.83333 | 0.76667 |  | 0.86667 | 0.93333 |
| mannose | 0.93333 | 0.8 | 0.56667 | 0.56667 | 0.53333 |
| mucic acid | 0.86667 | 0.73333 | 0.73333 | 0.76667 | 0.93333 |
| myo-inositol | 0.53333 | 0.81667 | 0.5 | 0.5 | 0.66667 |
| myristic acid |  |  | 0.56667 |  | 0.63333 |
| N-acetylaspartic acid | 0.66667 | 0.6 | 0.6 | 0.63333 | 0.8 |
| n-acetyl-d-hexosamine | 0.53333 | 0.83333 | 0.9 | 0.76667 | 0.7 |
| N-acetylglutamate |  |  |  |  | 0.7 |
| N-acetylmannosamine | 0.63333 | 0.73333 | 0.73333 | 0.66667 | 0.63333 |
| ornithine | 0.83333 | 0.7 | 0.76667 | 0.66667 | 0.7 |
| oxalic acid | 0.83333 | 0.76667 | 0.73333 | 0.7 | 0.93333 |
| oxoproline | 0.73333 | 0.96667 | 0.63333 | 0.9 | 0.76667 |
| palmitic acid | 0.56667 | 0.83333 | 0.73333 | 0.8 | 0.66667 |
| pelargonic acid | 0.53333 | 0.86667 | 0.53333 | 0.9 | 0.66667 |
| pentitol |  | 0.73333 |  | 0.53333 | 0.76667 |
| phenaceturic acid | 0.6 | 0.63333 | 0.53333 | 0.8 | 0.7 |
| phenol | 0.6 | 0.9 | 0.8 | 0.8 | 0.76667 |
| phosphate | 0.56667 | 0.56667 | 0.6 | 0.66667 | 0.73333 |
| pimelic acid | 0.8 | 0.96667 | 0.83333 | 0.8 | 0.96667 |
| pinitol | 0.7 | 0.8 | 0.7 | 0.93333 | 0.76667 |
| propane-1,3-diol NIST | 0.66667 | 0.7 | 0.5 | 0.6 | 0.56667 |
| pseudo uridine | 0.6 | 0.9 | 1 | 0.93333 | 0.9 |
| putrescine | 0.56667 | 1 | 0.96667 | 0.76667 | 0.76667 |
| pyruvic acid | 0.56667 | 0.83333 | 0.63333 | 0.5 | 0.6 |
| quinic acid | 0.73333 | 0.73333 | 0.53333 |  | 0.56667 |
| raffinose | 0.73333 | 0.73333 | 0.63333 | 0.86667 | 0.63333 |
| ribitol | 0.53333 | 0.86667 | 0.9 | 0.7 | 0.8 |
| ribonic acid | 0.7 | 0.83333 | 0.9 | 0.76667 | 0.53333 |
| ribose | 0.63333 | 0.83333 | 0.9 | 0.8 | 0.56667 |
| saccharic acid | 0.56667 | 0.56667 | 0.7 | 0.86667 | 0.73333 |
| serine | 0.56667 |  |  | 0.7 | 0.9 |
| sorbitol | 0.76667 | 0.76667 | 0.76667 | 0.53333 | 0.6 |
| stearic acid | 0.53333 | 0.83333 | 0.73333 | 0.66667 | 0.63333 |
| succinic acid |  | 0.66667 | 0.9 | 0.53333 | 0.76667 |
| sucrose | 0.53333 | 0.6 | 0.53333 | 0.73333 | 0.56667 |
| sulfuric acid | 0.53333 | 0.83333 | 0.5 | 0.53333 | 0.73333 |
| tagatose |  |  |  |  | 0.96667 |
| taurine | 0.66667 | 1 | 1 | 0.93333 | 0.93333 |
| threitol | 0.76667 | 0.8 | 0.8 | 0.83333 | 0.83333 |
| threonic acid | 0.73333 | 1 | 0.9 | 0.96667 | 0.86667 |
| trehalose | 0.6 | 0.83333 | 0.73333 | 0.76667 | 0.56667 |
| tryptophan | 0.75 | 1 | 0.9 | 0.96667 | 0.9 |
| tyrosine | 0.76667 | 0.93333 | 0.93333 | 1 | 1 |
| tyrosol | 0.96667 | 0.83333 | 0.63333 | 0.8 | 0.53333 |
| urea | 0.6 |  | 0.7 | 0.96667 | 0.76667 |
| uric acid | 0.56667 | 0.83333 | 0.9 | 0.83333 | 0.86667 |
| uridine | 0.73333 | 1 | 0.7 | 0.93333 | 0.66667 |
| valine | 0.66667 | 0.56667 | 0.76667 | 0.73333 | 0.53333 |
| vanillic acid | 0.76667 | 0.6 | 0.73333 | 0.7 | 0.63333 |
| xylitol | 0.56667 | 0.93333 | 0.96667 | 0.93333 | 0.86667 |
| xylonic acid | 0.8 |  |  | 0.93333 | 0.8 |
| xylose | 0.76667 | 0.76667 | 0.83333 | 0.66667 | 0.56667 |
| xylulose NIST | 0.5 | 0.93333 | 0.86667 | 0.9 | 0.76667 |
