# Supplementary material for: Urinary chemical fingerprint left behind by repeated NSAID administration: Discovery of putative biomarkers using artificial intelligence
Source: PLoS One. 2020 Feb 13;15(2):e0228989. doi: 10.1371/journal.pone.0228989 (PMC7018043; doi:10.1371/journal.pone.0228989)
Supplement: S4 Table — Tubule damage and inflammation were assessed on hematoxylin and eosin (H&E) stained sections and basement membrane integrity were assessed on periodic acid-Schiff methenamine silver (PAMS) stained sections. Cortical and corticomedullary tubules were assessed for degeneration, necrosis, regeneration, dilation, attenuation and hypertrophy. Any subtype of inflammatory cell within the interstitium (i.e. neutrophil, lymphocyte, macrophage, eosinophil, etc.) were counted when determining extent of inflammation. The maximum score per category is 4 with a maximum possible score of 16. (DOCX) [file pone.0228989.s011.docx]

**Supplemental Table S4:** Raw semi-quantitative renal histologic scores for the testing data set obtained from blinded examination of at least one kidney from each control cat treated with saline (n=4) or meloxicam (n=4) at 0.3 mg/kg every 24 hrs for 17 days. Tubule damage and inflammation were assessed on hematoxylin and eosin (H&E) stained sections and basement membrane integrity were assessed on periodic acid-Schiff methenamine silver (PAMS) stained sections. Cortical and corticomedullary tubules were assessed for degeneration, necrosis, regeneration, dilation, attenuation and hypertrophy. Any subtype of inflammatory cell within the interstitium (i.e. neutrophil, lymphocyte, macrophage, eosinophil, etc.) were counted when determining extent of inflammation. The maximum score per category is 4 with a maximum possible score of 16.

|  | **M1 LK** | **M1 RK** | **C2 LK** | **C2 RK** | **C3 LK** | **M4 LK** | **M4 RK** | **M5 LK** | **M5 RK** | **C6 LK** | **C7 LK** | **C8 LK** | **C8 RK** |
| --- | --- | --- | --- | --- | --- | --- | --- | --- | --- | --- | --- | --- | --- |
| **Tubules** |  |  |  |  |  |  |  |  |  |  |  |  |  |
| % Cortical tubular damage |  |  |  |  |  |  |  |  |  |  |  |  |  |
| No to minimal (<1%) |  |  |  | 0 | 0 |  |  |  |  |  |  | 0 | 0 |
| Scattered (1-25%) |  |  | 1 |  |  |  |  |  |  | 1 | 1 |  |  |
| Multifocal (26-50%) | 2 | 2 |  |  |  | 2 |  |  | 2 |  |  |  |  |
| Extensive (50-75%) |  |  |  |  |  |  | 3 | 3 |  |  |  |  |  |
| Diffuse (75-100%) |  |  |  |  |  |  |  |  |  |  |  |  |  |
|  |  |  |  |  |  |  |  |  |  |  |  |  |  |
| % Corticomedullary tubular damage |  |  |  |  |  |  |  |  |  |  |  |  |  |
| No to minimal (<1%) |  |  | 0 | 0 | 0 |  |  |  |  |  |  | 0 | 0 |
| Scattered (1-25%) |  |  |  |  |  |  |  |  |  | 1 | 1 |  |  |
| Multifocal (26-50%) | 2 | 2 |  |  |  |  |  |  |  |  |  |  |  |
| Extensive (50-75%) |  |  |  |  |  | 3 | 3 | 3 | 3 |  |  |  |  |
| Diffuse (75-100%) |  |  |  |  |  |  |  |  |  |  |  |  |  |
|  |  |  |  |  |  |  |  |  |  |  |  |  |  |
| % Normal basement membrane (BM) |  |  |  |  |  |  |  |  |  |  |  |  |  |
| No to minimal (<1%) | 0 |  | 0 | 0 | 0 |  | 0 |  |  | 0 | 0 | 0 | 0 |
| Scattered (1-25%) |  | 1 |  |  |  | 1 |  |  | 1 |  |  |  |  |
| Multifocal (26-50%) |  |  |  |  |  |  |  | 2 |  |  |  |  |  |
| Extensive (50-75%) |  |  |  |  |  |  |  |  |  |  |  |  |  |
| Diffuse (75-100%) |  |  |  |  |  |  |  |  |  |  |  |  |  |
|  |  |  |  |  |  |  |  |  |  |  |  |  |  |
| **Interstitium** |  |  |  |  |  |  |  |  |  |  |  |  |  |
| % Interstital Inflammation |  |  |  |  |  |  |  |  |  |  |  |  |  |
| No inflammation | 0 |  | 0 | 0 | 0 |  |  |  |  | 0 |  | 0 | 0 |
| <25% |  | 1 |  |  |  | 1 | 1 | 1 |  |  | 1 |  |  |
| 25-50% |  |  |  |  |  |  |  |  | 2 |  |  |  |  |
| 51-75% |  |  |  |  |  |  |  |  |  |  |  |  |  |
| >75% |  |  |  |  |  |  |  |  |  |  |  |  |  |
|  |  |  |  |  |  |  |  |  |  |  |  |  |  |
| **Total** | 4 | 6 | 1 | 0 | 0 | 7 | 7 | 9 | 8 | 2 | 3 | 0 | 0 |
|  | **M1 LK** | **M1 RK** | **M2 LK** | **M2 RK** | **C3 LK** | **M4 LK** | **M4 RK** | **M5 LK** | **M5 RK** | **C6 LK** | **C7 LK** | **C8 LK** | **C8 RK** |
