# Supplementary material for: Urinary chemical fingerprint left behind by repeated NSAID administration: Discovery of putative biomarkers using artificial intelligence
Source: PLoS One. 2020 Feb 13;15(2):e0228989. doi: 10.1371/journal.pone.0228989 (PMC7018043; doi:10.1371/journal.pone.0228989)
Supplement: S5 Table — The list of identified metabolites is derived from training data set urine collected from control cats treated with saline (n = 6) or meloxicam (n = 5) at 0.3 mg/kg every 24 hrs for up to 17 days (time point 5). Values highlighted in red indicate an increase in average intensity (> 1.2 fold) and values highlighted in yellow represent a decrease in average intensity (< 0.8) when comparing the meloxicam treated group vs. the saline treated control group. (DOCX) [file pone.0228989.s012.docx]

**Supplemental Table S5:** List of average, maximum, and minimum raw intensity values (m/z) obtained from GC-MS assay for each time point. The list of identified metabolites is derived from training data set urine collected from control cats treated with saline (n=6) or meloxicam (n=5) at 0.3 mg/kg every 24 hrs for up to 17 days (time point 5). Values highlighted in red indicate an increase in average intensity (> 1.2 fold) and values highlighted in yellow represent a decrease in average intensity (< 0.8) when comparing the meloxicam treated group vs. the saline treated control group.

| **Time 1** | **Average** | **Max** | **Min** | **Average** | **Max** | **Min** | **Ratio** |
| --- | --- | --- | --- | --- | --- | --- | --- |
| **Phenotype** | **M** | **M** | **M** | **C** | **C** | **C** | **M/C** |
| 3-(3-hydroxyphenyl)propionic acid | 55052.6 | 90841 | 35784 | 76646.83 | 123508 | 55159 | 0.718263 |
| 3-(4-hydroxyphenyl)propionic acid | 49632 | 73626 | 25680 | 58503.5 | 96516 | 11651 | 0.848359 |
| 3,4-dihydroxycinnamic acid | 542.6 | 940 | 256 | 607.5 | 1282 | 216 | 0.893169 |
| 3,4-dihydroxyhydrocinnamic acid NIST | 13650.8 | 34018 | 1898 | 3631.833 | 7816 | 1032 | 3.758653 |
| 3,4-dihydroxyphenylacetic acid | 6763.6 | 7851 | 4853 | 5757.833 | 7214 | 3927 | 1.174678 |
| 3-aminoisobutyric acid | 715 | 1580 | 139 | 17992.5 | 104115 | 550 | 0.039739 |
| 3-hydroxy-3-methylglutaric acid | 2633.6 | 3782 | 1924 | 1380.333 | 3276 | 521 | 1.907945 |
| 3-phosphoglycerate | 2736.2 | 3556 | 2222 | 3209.5 | 7641 | 456 | 0.852532 |
| 4-hydroxybenzoate | 688.6 | 1110 | 132 | 701.3333 | 1028 | 507 | 0.981844 |
| 4-hydroxybutyric acid | 2068.2 | 2618 | 1787 | 2697 | 3550 | 1970 | 0.766852 |
| 4-hydroxycinnamic acid | 786.6 | 882 | 703 | 884.5 | 1223 | 640 | 0.889316 |
| 4-hydroxyhippuric acid NIST | 8509.4 | 11342 | 4454 | 6389.833 | 16285 | 1491 | 1.331709 |
| 4-hydroxyphenylacetic acid | 28131.4 | 58191 | 17539 | 44418.17 | 66353 | 31913 | 0.633331 |
| 5-hydroxy-3-indoleacetic acid | 4651.4 | 6762 | 1693 | 5047.333 | 6816 | 3412 | 0.921556 |
| aconitic acid | 22802.6 | 26227 | 18222 | 20090.83 | 25176 | 17159 | 1.134975 |
| adenosine | 6730.4 | 9844 | 3117 | 7672.167 | 12756 | 898 | 0.877249 |
| alanine | 12527.4 | 20859 | 6835 | 19367.33 | 50800 | 9055 | 0.646831 |
| allantoic acid | 27449 | 66500 | 6508 | 26169 | 64813 | 7602 | 1.048913 |
| alpha-ketoglutarate | 9754.4 | 11995 | 5379 | 4927.833 | 6780 | 2729 | 1.97945 |
| benzoic acid | 13364 | 17218 | 9955 | 12787 | 17203 | 7738 | 1.045124 |
| benzylalcohol | 98428.8 | 144269 | 80021 | 120166 | 212191 | 62420 | 0.819107 |
| beta-alanine | 5980 | 16490 | 1088 | 9967.5 | 18830 | 3358 | 0.59995 |
| beta-gentiobiose | 5181 | 8473 | 2256 | 6287.333 | 10281 | 1876 | 0.824038 |
| catechol | 2051.8 | 4636 | 986 | 3158 | 5344 | 1508 | 0.649715 |
| citramalic acid | 1716 | 2336 | 911 | 1475.167 | 2237 | 1165 | 1.163258 |
| citric acid | 649732.6 | 956910 | 255319 | 200302 | 504516 | 84437 | 3.243765 |
| citrulline | 2619.8 | 3358 | 1910 | 4660.333 | 6919 | 3078 | 0.562149 |
| conduritol-beta-expoxide | 3626.4 | 4244 | 3078 | 3622 | 4760 | 2039 | 1.001215 |
| creatinine | 38001.6 | 79614 | 1080 | 83339.67 | 200412 | 2857 | 0.455985 |
| deoxypentitol | 2773.4 | 3451 | 2291 | 3138.833 | 4502 | 2289 | 0.883577 |
| erythritol | 76514.4 | 86594 | 58965 | 78876.33 | 97395 | 61793 | 0.970055 |
| ferulic acid | 560.8 | 1017 | 70 | 914 | 2036 | 346 | 0.613567 |
| fructose | 8243.4 | 10192 | 4736 | 10622.83 | 14138 | 5931 | 0.776008 |
| fucose | 64104.8 | 81816 | 56705 | 77903.33 | 112691 | 59003 | 0.822876 |
| galactinol | 11591.4 | 24651 | 1243 | 6045 | 26379 | 1115 | 1.917519 |
| galactonic acid | 1823.8 | 2520 | 1170 | 2205.333 | 2559 | 1650 | 0.826995 |
| gluconic acid | 3711 | 5478 | 2684 | 4227.833 | 4806 | 3487 | 0.877755 |
| glyceric acid | 17632.6 | 29927 | 6335 | 25038.17 | 44321 | 3056 | 0.704229 |
| glycerol | 37248 | 48463 | 29866 | 64388.5 | 134587 | 23828 | 0.578488 |
| glycerol-3-galactoside | 2173.4 | 3117 | 1029 | 3004 | 3958 | 2004 | 0.723502 |
| glycine | 11198.8 | 14007 | 7333 | 12228.17 | 15400 | 9508 | 0.91582 |
| glycocyamine | 13035.6 | 18655 | 4421 | 14957.33 | 34668 | 4401 | 0.871519 |
| glycolic acid | 1325.6 | 2004 | 826 | 1728.333 | 2190 | 936 | 0.766982 |
| hexadecane | 3389.2 | 4090 | 2015 | 3188.333 | 4514 | 2041 | 1.063001 |
| hexitol | 4062.8 | 5185 | 3337 | 4574.5 | 7215 | 2159 | 0.888141 |
| hexuronic acid | 2960 | 3661 | 1852 | 2995.333 | 3852 | 1872 | 0.988204 |
| hippuric acid | 63609 | 125883 | 32019 | 100922.5 | 196037 | 43993 | 0.630276 |
| hydroxylamine | 121166 | 153697 | 80185 | 138481.3 | 184486 | 82648 | 0.874963 |
| indole-3-acetate | 2134.4 | 4239 | 223 | 2197.167 | 7575 | 258 | 0.971433 |
| indoxyl sulfate | 35582.8 | 71697 | 8431 | 42288.17 | 81921 | 13952 | 0.841436 |
| inosine | 1187.6 | 2898 | 176 | 616 | 958 | 428 | 1.927922 |
| isocitric acid | 57921 | 71103 | 43856 | 49000.33 | 58911 | 41486 | 1.182053 |
| isohexonic acid | 9865.4 | 17261 | 6311 | 10184.5 | 15461 | 7272 | 0.968668 |
| isomaltose | 1045.8 | 1662 | 695 | 1124.667 | 1556 | 857 | 0.929876 |
| isoribose | 3937 | 5400 | 3091 | 4455.667 | 7160 | 2511 | 0.883594 |
| isothreonic acid | 30227 | 38012 | 21212 | 32725.33 | 37569 | 25316 | 0.923658 |
| kynurenic acid | 884.6 | 1191 | 583 | 958.5 | 1442 | 667 | 0.9229 |
| lactic acid | 2863.4 | 4068 | 1362 | 3365 | 5283 | 1994 | 0.850936 |
| lysine | 6800.8 | 10773 | 3285 | 9930.167 | 26284 | 1453 | 0.684863 |
| lyxitol | 51341.4 | 60735 | 40285 | 56402.17 | 77489 | 43299 | 0.910274 |
| lyxose | 2194.2 | 2827 | 1898 | 2787.5 | 4594 | 1347 | 0.787157 |
| malic acid | 857.8 | 1606 | 246 | 291.6667 | 495 | 97 | 2.941029 |
| mannose | 1818.6 | 2144 | 1606 | 2759.333 | 3714 | 2113 | 0.659072 |
| mucic acid | 1458 | 2665 | 302 | 2981.5 | 5401 | 1604 | 0.489016 |
| myo-inositol | 14402.2 | 17569 | 12607 | 16881.33 | 27005 | 12481 | 0.853144 |
| myristic acid | 543.2 | 588 | 507 | 622.1667 | 835 | 500 | 0.873078 |
| N-acetylaspartic acid | 6209.4 | 19678 | 1974 | 8664 | 40262 | 1773 | 0.71669 |
| n-acetyl-d-hexosamine | 4060.8 | 5541 | 2896 | 3956.667 | 5256 | 2867 | 1.026318 |
| N-acetylglutamate | 665.2 | 969 | 454 | 602.6667 | 712 | 443 | 1.103761 |
| N-acetylmannosamine | 2587.8 | 3250 | 1633 | 2316.333 | 3083 | 971 | 1.117197 |
| ornithine | 5360 | 8026 | 2615 | 2838.167 | 5772 | 1185 | 1.888543 |
| oxalic acid | 1755.2 | 2378 | 694 | 2841 | 3790 | 1810 | 0.617811 |
| oxoproline | 65919 | 75478 | 55711 | 90016.17 | 138668 | 54705 | 0.732302 |
| palmitic acid | 13053.4 | 15306 | 10424 | 13203.17 | 20349 | 9403 | 0.988657 |
| pelargonic acid | 5503.8 | 8963 | 3148 | 5370.5 | 7419 | 2303 | 1.024821 |
| pentitol | 470.6 | 601 | 339 | 494.6667 | 935 | 285 | 0.951348 |
| phenaceturic acid | 267709.4 | 360606 | 161801 | 283620.8 | 410522 | 153843 | 0.943899 |
| phenol | 20029 | 27173 | 10376 | 26352.33 | 49439 | 7666 | 0.760047 |
| phosphate | 547616.8 | 854081 | 144541 | 669001.8 | 804571 | 521300 | 0.818558 |
| pimelic acid | 1987.2 | 2391 | 1357 | 2615.667 | 3942 | 1775 | 0.75973 |
| pinitol | 30402.2 | 42443 | 16830 | 40917.33 | 66742 | 27511 | 0.743015 |
| propane-1,3-diol NIST | 3897.6 | 4962 | 2672 | 4735.5 | 8117 | 1063 | 0.82306 |
| pseudo uridine | 154616.2 | 182484 | 101064 | 179601.8 | 252008 | 115752 | 0.860883 |
| putrescine | 4844.6 | 5804 | 3123 | 4649.5 | 6821 | 3020 | 1.041962 |
| pyruvic acid | 8420.6 | 10287 | 6893 | 8651.833 | 16320 | 1837 | 0.973273 |
| quinic acid | 277.2 | 418 | 168 | 503.5 | 934 | 157 | 0.550546 |
| raffinose | 2172.4 | 4639 | 995 | 2792.333 | 3921 | 1358 | 0.777987 |
| ribitol | 9081.2 | 11966 | 7092 | 9472.333 | 14603 | 4474 | 0.958708 |
| ribonic acid | 3417.4 | 4238 | 2827 | 4010.5 | 5569 | 2940 | 0.852113 |
| ribose | 7664.2 | 9860 | 5789 | 8728.667 | 11829 | 5947 | 0.878049 |
| saccharic acid | 9696.8 | 17261 | 5225 | 9194 | 14833 | 1488 | 1.054688 |
| serine | 392 | 856 | 176 | 678.1667 | 2261 | 118 | 0.578029 |
| sorbitol | 10315.2 | 13405 | 8810 | 18320.83 | 23742 | 6200 | 0.563031 |
| stearic acid | 69405.4 | 82227 | 50345 | 71325.67 | 103945 | 51244 | 0.973077 |
| succinic acid | 820.6 | 1114 | 662 | 673.3333 | 1014 | 347 | 1.218713 |
| sucrose | 6179.2 | 14349 | 280 | 4763 | 9178 | 959 | 1.297334 |
| sulfuric acid | 77163.8 | 151541 | 32817 | 80074.67 | 181305 | 20049 | 0.963648 |
| tagatose | 545 | 620 | 490 | 671.1667 | 977 | 497 | 0.812019 |
| taurine | 134246.6 | 364545 | 42299 | 62028.17 | 144638 | 26806 | 2.164285 |
| threitol | 6967.8 | 8079 | 5328 | 8168.5 | 10198 | 5672 | 0.853009 |
| threonic acid | 4545 | 4690 | 4252 | 5258 | 7474 | 3739 | 0.864397 |
| trehalose | 3933.6 | 7048 | 859 | 4606.333 | 6154 | 2479 | 0.853955 |
| tryptophan | 1484.2 | 1781 | 1165 | 2562.333 | 5049 | 1378 | 0.579238 |
| tyrosine | 28275.6 | 52354 | 8933 | 45512 | 73703 | 29642 | 0.621278 |
| tyrosol | 1713 | 2243 | 1377 | 4327.5 | 6685 | 2114 | 0.395841 |
| urea | 2358558 | 5173121 | 155030 | 2434405 | 5194415 | 87595 | 0.968844 |
| uric acid | 20254.6 | 43046 | 9445 | 22136.5 | 48417 | 12547 | 0.914987 |
| uridine | 1606.4 | 2034 | 1194 | 2155.333 | 3109 | 1146 | 0.745314 |
| valine | 1709.8 | 2472 | 1141 | 2440 | 5608 | 1413 | 0.700738 |
| vanillic acid | 2083.6 | 2658 | 1484 | 1546.833 | 3093 | 755 | 1.34701 |
| xylitol | 12107.8 | 13604 | 9066 | 14323 | 19692 | 7845 | 0.84534 |
| xylonic acid | 343 | 506 | 142 | 564.8333 | 999 | 190 | 0.607259 |
| xylose | 23820.8 | 33367 | 12580 | 31375.5 | 46935 | 21327 | 0.759217 |
| xylulose NIST | 1942.8 | 3867 | 879 | 1919.167 | 3942 | 366 | 1.012314 |

| Time 2 | Average | Max | Min | Average | Max | Min | Ratio |
| --- | --- | --- | --- | --- | --- | --- | --- |
| Phenotype | M | M | M | C | C | C | M/C |
| 3-(3-hydroxyphenyl)propionic acid | 41575.6 | 69339 | 830 | 55669.83 | 107125 | 9572 | 0.746825 |
| 3-(4-hydroxyphenyl)propionic acid | 35072.8 | 66320 | 10797 | 39194.83 | 90492 | 20419 | 0.894832 |
| 3,4-dihydroxycinnamic acid | 560 | 1360 | 206 | 1172.833 | 3343 | 294 | 0.477476 |
| 3,4-dihydroxyhydrocinnamic acid NIST | 14511.8 | 50558 | 1096 | 11101.33 | 35434 | 1526 | 1.307212 |
| 3,4-dihydroxyphenylacetic acid | 6239.6 | 7573 | 4551 | 6706.833 | 8357 | 4127 | 0.930335 |
| 3-aminoisobutyric acid | 1034 | 1677 | 266 | 10267.33 | 57213 | 500 | 0.100708 |
| 3-hydroxy-3-methylglutaric acid | 2329.4 | 3332 | 1323 | 1173.5 | 3386 | 276 | 1.985002 |
| 3-phosphoglycerate | 1467.2 | 1680 | 1280 | 1894.333 | 3639 | 927 | 0.77452 |
| 4-hydroxybenzoate | 871.4 | 1622 | 540 | 488.1667 | 914 | 47 | 1.785046 |
| 4-hydroxybutyric acid | 4257.2 | 5154 | 2992 | 2348.333 | 4257 | 1349 | 1.81286 |
| 4-hydroxycinnamic acid | 642.6 | 1059 | 354 | 918 | 1382 | 601 | 0.7 |
| 4-hydroxyhippuric acid NIST | 7881.8 | 10100 | 6355 | 5909.167 | 8878 | 2732 | 1.333826 |
| 4-hydroxyphenylacetic acid | 23830 | 35497 | 10712 | 35992.5 | 48208 | 20942 | 0.662082 |
| 5-hydroxy-3-indoleacetic acid | 3686.8 | 6515 | 1853 | 5452.833 | 6877 | 3167 | 0.676126 |
| aconitic acid | 18484.8 | 22539 | 13408 | 19314 | 23209 | 15672 | 0.957067 |
| adenosine | 2646.2 | 5010 | 112 | 7562 | 11885 | 2184 | 0.349934 |
| alanine | 11901.6 | 16185 | 8541 | 16372.5 | 22912 | 9034 | 0.726926 |
| allantoic acid | 8651.8 | 10908 | 1684 | 15393.17 | 41245 | 7602 | 0.562055 |
| alpha-ketoglutarate | 9786 | 16706 | 6519 | 5275.167 | 9975 | 3002 | 1.855107 |
| benzoic acid | 18901.2 | 23598 | 13452 | 14930 | 27817 | 8062 | 1.265988 |
| beta-alanine | 11862.6 | 20316 | 3252 | 9559 | 16840 | 4821 | 1.240988 |
| beta-gentiobiose | 3395 | 5996 | 840 | 9139.333 | 15000 | 2608 | 0.371471 |
| catechol | 1524.4 | 2921 | 134 | 2253.167 | 4219 | 627 | 0.676559 |
| citramalic acid | 1841.4 | 3470 | 1301 | 1286 | 1600 | 891 | 1.431882 |
| citric acid | 871486.8 | 1222824 | 319938 | 185115 | 425765 | 55672 | 4.707813 |
| citrulline | 2737 | 5145 | 171 | 4781.333 | 6471 | 2621 | 0.572434 |
| conduritol-beta-expoxide | 2127 | 3070 | 187 | 3919.5 | 7149 | 2842 | 0.542671 |
| creatinine | 27362.4 | 82448 | 5563 | 73842 | 124492 | 10621 | 0.370553 |
| deoxypentitol | 2233 | 3219 | 1398 | 3392.667 | 4502 | 2586 | 0.658184 |
| erythritol | 67819.8 | 85493 | 48689 | 81383.5 | 100419 | 52920 | 0.833336 |
| ferulic acid | 558.4 | 1745 | 51 | 1169.667 | 3579 | 169 | 0.477401 |
| fructose | 6099.6 | 9585 | 4023 | 13031 | 23289 | 8271 | 0.468084 |
| fucose | 53382.2 | 79739 | 30492 | 81171.33 | 112691 | 54397 | 0.657648 |
| galactinol | 4509.4 | 14891 | 1039 | 10998.5 | 31313 | 2022 | 0.410001 |
| galactonic acid | 2069.6 | 3383 | 598 | 2884.833 | 4681 | 1863 | 0.717407 |
| gluconic acid | 2851.2 | 3883 | 1874 | 4164.667 | 5200 | 2690 | 0.684617 |
| glyceric acid | 13444.8 | 29953 | 4164 | 20225.5 | 30131 | 1433 | 0.664745 |
| glycerol | 36991.8 | 47144 | 24323 | 59033 | 129807 | 28904 | 0.626629 |
| glycerol-3-galactoside | 2345.8 | 3664 | 1242 | 3207.333 | 3966 | 2186 | 0.731386 |
| glycine | 35940.4 | 109967 | 13658 | 13147.5 | 24824 | 8218 | 2.73363 |
| glycocyamine | 2237.8 | 5022 | 208 | 17084.5 | 29496 | 5626 | 0.130984 |
| glycolic acid | 1495.8 | 2953 | 0 | 1839.833 | 3571 | 1050 | 0.813008 |
| hexadecane | 4451 | 7340 | 1983 | 3466.667 | 7881 | 2018 | 1.283942 |
| hexitol | 3593 | 4416 | 2031 | 5623.667 | 9040 | 3525 | 0.638907 |
| hexuronic acid | 2008.6 | 3172 | 1018 | 3163.333 | 4331 | 1777 | 0.634963 |
| hippuric acid | 37500.8 | 76044 | 10266 | 91982 | 192506 | 28097 | 0.407697 |
| hydroxylamine | 184982.6 | 285586 | 111632 | 130433.2 | 252697 | 68528 | 1.418218 |
| indole-3-acetate | 1201.6 | 4124 | 394 | 1254.167 | 2988 | 654 | 0.958086 |
| indoxyl sulfate | 31298.2 | 65890 | 3995 | 57470.83 | 112178 | 8029 | 0.544593 |
| inosine | 875.2 | 2337 | 267 | 726.6667 | 1977 | 339 | 1.204404 |
| isocitric acid | 45655.4 | 57428 | 27356 | 46422.83 | 55551 | 36027 | 0.983469 |
| isohexonic acid | 6023.8 | 18588 | 515 | 6985.167 | 13244 | 1243 | 0.86237 |
| isomaltose | 1076.6 | 1977 | 384 | 1320.5 | 2083 | 787 | 0.815297 |
| isoribose | 3639.2 | 4946 | 2655 | 4494.333 | 5051 | 3898 | 0.809731 |
| isothreonic acid | 24582.4 | 33545 | 16022 | 33549.67 | 39441 | 18341 | 0.732717 |
| kynurenic acid | 445.4 | 723 | 112 | 870.3333 | 1188 | 297 | 0.511758 |
| lactic acid | 5270 | 9922 | 2906 | 3801.167 | 7593 | 1570 | 1.386416 |
| lysine | 1063.4 | 2620 | 158 | 10212.5 | 16565 | 2040 | 0.104127 |
| lyxitol | 45226.4 | 55881 | 32728 | 62530.5 | 77489 | 48601 | 0.723269 |
| lyxose | 8150 | 31815 | 1504 | 2953.167 | 3810 | 1908 | 2.759749 |
| malic acid | 1063 | 2606 | 323 | 356.6667 | 625 | 126 | 2.980374 |
| mannose | 1985.2 | 3680 | 1271 | 2633 | 2990 | 1958 | 0.753969 |
| mucic acid | 2051.2 | 6023 | 472 | 3107.167 | 4448 | 858 | 0.660151 |
| myo-inositol | 11712.2 | 15327 | 5958 | 15380.83 | 18508 | 9703 | 0.76148 |
| myristic acid | 648.8 | 1094 | 43 | 565.1667 | 1001 | 350 | 1.14798 |
| N-acetylaspartic acid | 4821.8 | 14535 | 2090 | 10726.17 | 52888 | 1368 | 0.449536 |
| n-acetyl-d-hexosamine | 2873 | 4058 | 2508 | 4913.167 | 6363 | 2334 | 0.584755 |
| N-acetylglutamate | 439.6 | 538 | 360 | 593.1667 | 767 | 411 | 0.741107 |
| N-acetylmannosamine | 2119.2 | 2464 | 1642 | 2638.833 | 3353 | 1703 | 0.803082 |
| ornithine | 6474 | 10204 | 1096 | 2845.333 | 4772 | 1185 | 2.275305 |
| oxalic acid | 1989.8 | 2897 | 1498 | 2694.833 | 4899 | 775 | 0.738376 |
| oxoproline | 55837.8 | 93309 | 31210 | 108380.5 | 128180 | 89781 | 0.515202 |
| palmitic acid | 18445.8 | 25196 | 13010 | 12289.83 | 27647 | 7504 | 1.500899 |
| pelargonic acid | 7244 | 10897 | 4367 | 4452.167 | 9255 | 2303 | 1.627073 |
| pentitol | 434.4 | 833 | 228 | 704.6667 | 1564 | 285 | 0.616462 |
| phenaceturic acid | 231006.4 | 361702 | 144654 | 274259.3 | 374038 | 96686 | 0.842292 |
| phenol | 5044.4 | 14452 | 211 | 25618.5 | 43657 | 3913 | 0.196905 |
| phosphate | 765950.2 | 1217603 | 359882 | 697357.8 | 919020 | 521040 | 1.09836 |
| pimelic acid | 1342.4 | 2227 | 918 | 2701.833 | 3084 | 1794 | 0.496848 |
| pinitol | 21719.4 | 47714 | 9645 | 38975.83 | 44245 | 30593 | 0.557253 |
| propane-1,3-diol NIST | 6221.6 | 9800 | 3338 | 5111.167 | 11991 | 2553 | 1.217256 |
| pseudo uridine | 96673 | 170822 | 24573 | 190085.3 | 222148 | 102301 | 0.508577 |
| putrescine | 1240.4 | 2295 | 483 | 4997.5 | 5967 | 4129 | 0.248204 |
| pyruvic acid | 4000.6 | 7589 | 147 | 8582.167 | 15137 | 4712 | 0.466153 |
| quinic acid | 496.2 | 1110 | 115 | 849.5 | 1291 | 223 | 0.584108 |
| raffinose | 2065.4 | 4910 | 292 | 4082.5 | 8838 | 700 | 0.505915 |
| ribitol | 7190 | 9510 | 5851 | 11632.5 | 17951 | 7009 | 0.618096 |
| ribonic acid | 2635 | 4432 | 35 | 4908.5 | 7186 | 2959 | 0.536824 |
| ribose | 5816.6 | 8381 | 3923 | 8947 | 11829 | 5607 | 0.650117 |
| saccharic acid | 8911 | 18325 | 3517 | 8629 | 16241 | 1439 | 1.03268 |
| serine | 213.8 | 528 | 57 | 432.3333 | 710 | 263 | 0.494526 |
| sorbitol | 11273.2 | 20918 | 4409 | 16457.33 | 23599 | 6381 | 0.684996 |
| stearic acid | 104981.6 | 165730 | 67853 | 72206.5 | 184814 | 42015 | 1.453908 |
| succinic acid | 854.4 | 1527 | 60 | 676 | 1029 | 377 | 1.263905 |
| sucrose | 5491.2 | 10389 | 2208 | 8343.833 | 19979 | 1518 | 0.658115 |
| sulfuric acid | 30256.2 | 44845 | 25635 | 64999 | 111725 | 9942 | 0.465487 |
| tagatose | 456 | 649 | 387 | 674.1667 | 861 | 548 | 0.676391 |
| taurine | 9489.2 | 46064 | 44 | 109299.8 | 213640 | 64594 | 0.086818 |
| threitol | 6637 | 10522 | 4039 | 8716.667 | 10769 | 6638 | 0.761415 |
| threonic acid | 3470.4 | 5033 | 1593 | 6219.167 | 7474 | 5273 | 0.558017 |
| trehalose | 3850.4 | 7708 | 1643 | 5661.833 | 8609 | 4197 | 0.680062 |
| tryptophan | 864.8 | 1653 | 458 | 3162.667 | 5682 | 1779 | 0.27344 |
| tyrosine | 21384 | 39585 | 5254 | 44557.17 | 60335 | 34355 | 0.479923 |
| tyrosol | 1563 | 2325 | 995 | 3469.5 | 6644 | 1140 | 0.450497 |
| uric acid | 9583.4 | 32126 | 495 | 23106.5 | 44102 | 16732 | 0.414749 |
| uridine | 973 | 1266 | 684 | 2231.5 | 3109 | 1451 | 0.43603 |
| valine | 1829 | 3559 | 795 | 1754.333 | 2533 | 1412 | 1.042561 |
| vanillic acid | 1532.2 | 2130 | 953 | 1492 | 2509 | 852 | 1.026944 |
| xylitol | 7016.4 | 10839 | 3167 | 13873.5 | 16893 | 9090 | 0.505741 |
| xylonic acid | 548.8 | 813 | 330 | 708.3333 | 999 | 496 | 0.774776 |
| xylose | 26445.2 | 35184 | 20378 | 37658.83 | 46935 | 16950 | 0.702231 |
| xylulose NIST | 819.8 | 1187 | 31 | 1579.833 | 2834 | 1068 | 0.518915 |

| **Time 3** | **Average** | **Max** | **Min** | **Average** | **Max** | **Min** | **Ratio** |
| --- | --- | --- | --- | --- | --- | --- | --- |
| **Phenotype** | **M** | **M** | **M** | **C** | **C** | **C** | **M/C** |
| 3-(3-hydroxyphenyl)propionic acid | 27806.6 | 47598 | 6788 | 65922.33 | 149391 | 19320 | 0.421808 |
| 3-(4-hydroxyphenyl)propionic acid | 18748.6 | 41570 | 3102 | 50324.83 | 103391 | 21135 | 0.372552 |
| 3,4-dihydroxycinnamic acid | 419.8 | 480 | 230 | 1101.833 | 2595 | 422 | 0.381001 |
| 3,4-dihydroxyhydrocinnamic acid NIST | 3790.6 | 11408 | 599 | 15078 | 68772 | 1669 | 0.251399 |
| 3,4-dihydroxyphenylacetic acid | 5076.8 | 6957 | 559 | 7036.167 | 8966 | 4326 | 0.721529 |
| 3-aminoisobutyric acid | 831.2 | 1928 | 304 | 5965.5 | 31707 | 569 | 0.139335 |
| 3-hydroxy-3-methylglutaric acid | 2152.4 | 3062 | 828 | 1252.333 | 3250 | 405 | 1.718712 |
| 3-phosphoglycerate | 1064.6 | 2380 | 90 | 2561.167 | 7102 | 354 | 0.41567 |
| 4-hydroxybenzoate | 890.4 | 2252 | 263 | 639.8333 | 840 | 463 | 1.391612 |
| 4-hydroxybutyric acid | 4921.8 | 14015 | 1616 | 2385.667 | 3091 | 1605 | 2.063071 |
| 4-hydroxycinnamic acid | 596.6 | 788 | 380 | 992.5 | 1777 | 454 | 0.601108 |
| 4-hydroxyhippuric acid NIST | 4887.2 | 6992 | 983 | 6346.667 | 10500 | 2115 | 0.770042 |
| 4-hydroxyphenylacetic acid | 46903.2 | 119267 | 6142 | 28679.5 | 55649 | 18033 | 1.635426 |
| 5-hydroxy-3-indoleacetic acid | 3562 | 5262 | 2220 | 6134 | 9299 | 3591 | 0.580698 |
| aconitic acid | 15928.2 | 21613 | 4727 | 21703.5 | 27993 | 15771 | 0.7339 |
| adenosine | 992 | 1417 | 87 | 7365.667 | 15004 | 2432 | 0.134679 |
| alanine | 22846.8 | 45087 | 13238 | 16666.83 | 32276 | 9603 | 1.370794 |
| allantoic acid | 10301.4 | 21471 | 4170 | 20394.5 | 61580 | 6159 | 0.505107 |
| alpha-ketoglutarate | 8089.8 | 12041 | 1931 | 4976 | 7228 | 2201 | 1.625764 |
| benzoic acid | 19451.8 | 37427 | 11402 | 15023.67 | 21272 | 9366 | 1.294744 |
| benzylalcohol | 61311.2 | 154943 | 5442 | 106293 | 250039 | 38117 | 0.576813 |
| beta-alanine | 9643 | 26050 | 3144 | 7929 | 11710 | 4308 | 1.216168 |
| beta-gentiobiose | 6240.2 | 13717 | 1090 | 8999 | 16417 | 1670 | 0.693433 |
| catechol | 1517 | 3037 | 429 | 2665.833 | 6569 | 466 | 0.569053 |
| citramalic acid | 1613.2 | 1974 | 1153 | 1442.5 | 3309 | 490 | 1.118336 |
| citric acid | 613724.6 | 881953 | 121322 | 236978 | 485558 | 67167 | 2.589796 |
| citrulline | 2510.8 | 4656 | 854 | 6718 | 13575 | 2759 | 0.373742 |
| conduritol-beta-expoxide | 4117.6 | 7085 | 1329 | 4354.667 | 6255 | 2801 | 0.94556 |
| creatinine | 15871 | 37348 | 4823 | 56581.33 | 96533 | 34295 | 0.280499 |
| deoxypentitol | 2162.6 | 2436 | 1673 | 2940.167 | 3681 | 2161 | 0.735537 |
| erythritol | 66263.4 | 81131 | 45371 | 73977.67 | 91978 | 62323 | 0.895722 |
| ferulic acid | 341 | 574 | 133 | 868.5 | 1927 | 148 | 0.392631 |
| fructose | 8829.8 | 15212 | 4072 | 12350.17 | 16117 | 9684 | 0.714954 |
| fucose | 50998.8 | 73590 | 29168 | 78998.83 | 99007 | 48585 | 0.645564 |
| galactinol | 6475.2 | 21463 | 967 | 11412.17 | 24199 | 1708 | 0.567394 |
| galactonic acid | 2280.2 | 3191 | 1696 | 2510.833 | 3506 | 1737 | 0.908145 |
| gluconic acid | 3096.2 | 4154 | 1313 | 4693.5 | 6710 | 3254 | 0.659678 |
| glyceric acid | 9762.6 | 24087 | 1628 | 12434.83 | 30951 | 2251 | 0.785101 |
| glycerol | 50172 | 77922 | 37306 | 56394.33 | 104445 | 22757 | 0.889664 |
| glycerol-3-galactoside | 1273.2 | 1624 | 430 | 2703.333 | 3202 | 2213 | 0.470974 |
| glycine | 21194.4 | 47812 | 9558 | 13376.5 | 18547 | 9074 | 1.58445 |
| glycocyamine | 1792.4 | 3806 | 562 | 19560.5 | 38946 | 4727 | 0.091634 |
| glycolic acid | 1933.2 | 3081 | 687 | 2004.667 | 2552 | 1194 | 0.96435 |
| hexadecane | 5025 | 7882 | 2546 | 3494.667 | 5897 | 2138 | 1.437905 |
| hexitol | 5536.8 | 9726 | 1934 | 6274.333 | 8143 | 4598 | 0.882452 |
| hexuronic acid | 3049.8 | 3850 | 2300 | 3015.667 | 4299 | 2011 | 1.011319 |
| hippuric acid | 65501 | 147236 | 22279 | 113709 | 195796 | 42905 | 0.576041 |
| hydroxylamine | 230202.2 | 544135 | 91430 | 146217.2 | 188631 | 90933 | 1.574386 |
| indole-3-acetate | 1995.2 | 5922 | 235 | 3748.167 | 10010 | 679 | 0.532314 |
| indoxyl sulfate | 56324.4 | 103429 | 4291 | 47231 | 77193 | 29204 | 1.19253 |
| inosine | 550.6 | 832 | 90 | 667.8333 | 1327 | 289 | 0.824457 |
| isocitric acid | 37082.8 | 53848 | 4270 | 48759.17 | 63513 | 32285 | 0.76053 |
| isohexonic acid | 4832.8 | 8734 | 1077 | 6162.5 | 17238 | 843 | 0.784227 |
| isomaltose | 944.8 | 1859 | 304 | 927.1667 | 1537 | 535 | 1.019019 |
| isoribose | 2824.4 | 4123 | 1168 | 4432 | 5859 | 3381 | 0.637274 |
| isothreonic acid | 26774.2 | 33673 | 16543 | 32184.67 | 42098 | 29029 | 0.831893 |
| kynurenic acid | 571.4 | 757 | 329 | 1078 | 1526 | 651 | 0.530056 |
| lactic acid | 19267 | 77348 | 3132 | 3364.167 | 6556 | 659 | 5.727124 |
| lysine | 348.6 | 1205 | 69 | 15480.83 | 35080 | 1275 | 0.022518 |
| lyxitol | 41099 | 44813 | 36547 | 63928.83 | 71760 | 49160 | 0.642887 |
| lyxose | 2599.4 | 5298 | 1242 | 3324.333 | 5138 | 2235 | 0.781931 |
| malic acid | 620.8 | 734 | 386 | 369.8333 | 612 | 108 | 1.678594 |
| mannose | 2849 | 3940 | 2097 | 3006.833 | 3966 | 2149 | 0.947508 |
| mucic acid | 1389.2 | 3100 | 423 | 2884.667 | 6037 | 429 | 0.481581 |
| myo-inositol | 17311 | 23710 | 12819 | 17391 | 26442 | 11343 | 0.9954 |
| myristic acid | 874.2 | 1866 | 255 | 595.1667 | 893 | 288 | 1.468832 |
| N-acetylaspartic acid | 6197.8 | 23328 | 510 | 10628.67 | 51404 | 1593 | 0.583121 |
| n-acetyl-d-hexosamine | 2812.8 | 4082 | 664 | 5116.5 | 7659 | 3435 | 0.549751 |
| N-acetylglutamate | 403.6 | 549 | 167 | 544.6667 | 1037 | 117 | 0.741004 |
| N-acetylmannosamine | 1478.8 | 2395 | 585 | 2420.667 | 4475 | 1354 | 0.610906 |
| ornithine | 5536.4 | 7793 | 1137 | 3432.833 | 5962 | 1620 | 1.612779 |
| oxalic acid | 2067.6 | 3088 | 1151 | 2725.667 | 3612 | 1796 | 0.758567 |
| oxoproline | 80492.6 | 100408 | 50005 | 101024.2 | 150011 | 50073 | 0.796766 |
| palmitic acid | 20634.2 | 34022 | 11296 | 12740 | 18344 | 6795 | 1.619639 |
| pelargonic acid | 6515.8 | 16719 | 146 | 4672.167 | 7855 | 2412 | 1.394599 |
| pentitol | 376.8 | 837 | 122 | 427.6667 | 808 | 220 | 0.88106 |
| phenaceturic acid | 350729.8 | 708755 | 76837 | 242708.5 | 295769 | 129980 | 1.445066 |
| phenol | 6055.2 | 10589 | 1718 | 23785.33 | 59746 | 3396 | 0.254577 |
| phosphate | 762585.2 | 1193374 | 263561 | 625528.3 | 1149426 | 247127 | 1.219106 |
| pimelic acid | 1562.6 | 2043 | 1272 | 2542.333 | 4096 | 1096 | 0.614632 |
| pinitol | 22447.6 | 45333 | 3487 | 37491.33 | 75841 | 11242 | 0.598741 |
| propane-1,3-diol NIST | 7980.8 | 18310 | 2607 | 5690.833 | 8673 | 3282 | 1.402396 |
| pseudo uridine | 74831.6 | 94575 | 44055 | 200174.8 | 276352 | 151594 | 0.373831 |
| putrescine | 1222.4 | 2750 | 362 | 4635 | 6574 | 2078 | 0.263732 |
| pyruvic acid | 6268.2 | 9371 | 4391 | 8797.333 | 17626 | 2833 | 0.712511 |
| quinic acid | 577.4 | 1036 | 81 | 649.8333 | 978 | 109 | 0.888536 |
| raffinose | 1948.6 | 3915 | 339 | 4121.5 | 8338 | 285 | 0.472789 |
| ribitol | 5416 | 7825 | 2104 | 10898.33 | 16705 | 4557 | 0.496957 |
| ribonic acid | 3189.8 | 4315 | 2358 | 5007.667 | 7158 | 3377 | 0.636983 |
| ribose | 6383.2 | 8095 | 4554 | 9267.667 | 11840 | 6002 | 0.68876 |
| saccharic acid | 8790.6 | 18212 | 3344 | 13322.33 | 20641 | 4959 | 0.659839 |
| serine | 245.8 | 293 | 145 | 428.1667 | 545 | 146 | 0.574076 |
| sorbitol | 15261.6 | 20847 | 3331 | 10310.5 | 16884 | 6193 | 1.4802 |
| stearic acid | 113543.6 | 206293 | 58063 | 72396.17 | 97541 | 42232 | 1.568365 |
| succinic acid | 1158 | 1367 | 865 | 752.3333 | 1223 | 202 | 1.539211 |
| sucrose | 4775.2 | 9257 | 1117 | 4871.833 | 11743 | 1233 | 0.980165 |
| sulfuric acid | 53281.8 | 80378 | 23684 | 64536.67 | 130926 | 20153 | 0.825605 |
| tagatose | 478.8 | 636 | 300 | 663.1667 | 858 | 553 | 0.72199 |
| taurine | 84 | 159 | 18 | 118112.3 | 295335 | 2127 | 0.000711 |
| threitol | 6520.4 | 7461 | 5304 | 7579.667 | 9234 | 5939 | 0.860249 |
| threonic acid | 4680.6 | 5536 | 3176 | 6289.667 | 8744 | 5152 | 0.744173 |
| trehalose | 3398.8 | 6577 | 1230 | 5233.167 | 8801 | 1603 | 0.649473 |
| tryptophan | 1287.4 | 3179 | 488 | 3325 | 4143 | 2707 | 0.387188 |
| tyrosine | 21128 | 38195 | 12689 | 45529.67 | 79519 | 27234 | 0.464049 |
| tyrosol | 2334.8 | 5744 | 726 | 2562 | 5364 | 1426 | 0.911319 |
| urea | 3674114 | 6738821 | 63996 | 2050658 | 6561821 | 12918 | 1.791675 |
| uric acid | 6966.6 | 21756 | 467 | 26761.17 | 41247 | 12757 | 0.260325 |
| uridine | 1603.8 | 2020 | 794 | 2037.5 | 2605 | 1659 | 0.787141 |
| valine | 5361.6 | 17819 | 1834 | 1705.667 | 2550 | 830 | 3.143404 |
| vanillic acid | 968 | 1797 | 362 | 1394.167 | 2099 | 538 | 0.694322 |
| xylitol | 8615.6 | 12328 | 1681 | 14436 | 17155 | 10195 | 0.596814 |
| xylonic acid | 370.6 | 542 | 191 | 521 | 887 | 125 | 0.711324 |
| xylose | 26993.4 | 40312 | 20177 | 37374 | 42321 | 29286 | 0.722251 |
| xylulose NIST | 1267.4 | 2006 | 408 | 2147.667 | 3459 | 1083 | 0.590129 |

| **Time 4** | **Average** | **Max** | **Min** | **Average** | **Max** | **Min** | **Ratio** |
| --- | --- | --- | --- | --- | --- | --- | --- |
| **Phenotype** | **M** | **M** | **M** | **C** | **C** | **C** | **M/C** |
| 3-(3-hydroxyphenyl)propionic acid | 45441.6 | 75947 | 12992 | 55908 | 113423 | 28239 | 0.812792 |
| 3-(4-hydroxyphenyl)propionic acid | 30479.4 | 51265 | 2069 | 42917 | 72065 | 21448 | 0.710194 |
| 3,4-dihydroxycinnamic acid | 657.4 | 927 | 364 | 2247.167 | 3362 | 393 | 0.292546 |
| 3,4-dihydroxyhydrocinnamic acid NIST | 2176.4 | 2812 | 1569 | 7838.167 | 26086 | 1851 | 0.277667 |
| 3,4-dihydroxyphenylacetic acid | 5587 | 7194 | 3345 | 6005.167 | 7961 | 1574 | 0.930366 |
| 3-aminoisobutyric acid | 1872.4 | 4410 | 328 | 9041.167 | 50867 | 431 | 0.207097 |
| 3-hydroxy-3-methylglutaric acid | 1673.4 | 2685 | 475 | 1364.833 | 3415 | 296 | 1.226084 |
| 3-phosphoglycerate | 1740.4 | 3591 | 232 | 3040.833 | 6967 | 1062 | 0.572343 |
| 4-hydroxybenzoate | 1213.2 | 2681 | 276 | 658.8333 | 804 | 475 | 1.841437 |
| 4-hydroxybutyric acid | 4298.8 | 9683 | 1759 | 1925.5 | 2715 | 1248 | 2.232563 |
| 4-hydroxycinnamic acid | 1053.2 | 2432 | 523 | 1052.333 | 1527 | 394 | 1.000824 |
| 4-hydroxyhippuric acid NIST | 6364.8 | 9832 | 3339 | 7936.833 | 12366 | 4353 | 0.801932 |
| 4-hydroxyphenylacetic acid | 30056.2 | 65968 | 3688 | 29320.83 | 73479 | 8875 | 1.02508 |
| 5-hydroxy-3-indoleacetic acid | 2757.8 | 4226 | 1749 | 4624.5 | 5718 | 3970 | 0.596346 |
| aconitic acid | 13288.8 | 20404 | 4209 | 21361.5 | 30016 | 14588 | 0.622091 |
| adenosine | 1475.2 | 5398 | 145 | 5742.667 | 10869 | 2290 | 0.256884 |
| alanine | 19745 | 40680 | 11282 | 18404.17 | 34607 | 5918 | 1.072855 |
| allantoic acid | 11766.8 | 24852 | 2540 | 16265.33 | 47145 | 1447 | 0.723428 |
| alpha-ketoglutarate | 6850.4 | 12396 | 2514 | 4536.333 | 9378 | 1999 | 1.510118 |
| benzoic acid | 18013.8 | 27264 | 11311 | 11030.33 | 12486 | 9196 | 1.633115 |
| benzylalcohol | 69876.8 | 130967 | 8443 | 97827.67 | 155781 | 43502 | 0.714285 |
| beta-alanine | 24994 | 63798 | 3309 | 7185.5 | 14443 | 3367 | 3.478394 |
| beta-gentiobiose | 6962.2 | 9582 | 4059 | 16173.17 | 32324 | 5839 | 0.430478 |
| catechol | 2333.2 | 6057 | 688 | 3060 | 4631 | 744 | 0.762484 |
| citramalic acid | 1310.6 | 2926 | 566 | 1132.833 | 1589 | 903 | 1.156922 |
| citric acid | 601078.2 | 1418475 | 129938 | 232628.3 | 467586 | 71657 | 2.583856 |
| citrulline | 2454.4 | 3415 | 1012 | 6347.333 | 13359 | 2662 | 0.386682 |
| conduritol-beta-expoxide | 3597.2 | 5204 | 1921 | 5069.5 | 11443 | 3282 | 0.709577 |
| creatinine | 19900.2 | 43615 | 5252 | 54061.67 | 117871 | 10507 | 0.368102 |
| deoxypentitol | 2365.2 | 3887 | 1421 | 3132.5 | 3689 | 2185 | 0.755052 |
| erythritol | 63609.2 | 82426 | 34756 | 82198.17 | 118122 | 63639 | 0.773852 |
| ferulic acid | 529.2 | 746 | 368 | 2025.167 | 3099 | 424 | 0.261312 |
| fructose | 6653 | 11673 | 1259 | 15021.17 | 25518 | 8067 | 0.442908 |
| fucose | 39065 | 59645 | 17434 | 63794 | 90564 | 31197 | 0.612362 |
| galactinol | 5727 | 13068 | 1845 | 10532 | 29087 | 3603 | 0.543771 |
| galactonic acid | 1519.2 | 2187 | 892 | 3292.333 | 4606 | 1910 | 0.461436 |
| gluconic acid | 2795 | 3637 | 2492 | 3324 | 5668 | 412 | 0.840854 |
| glyceric acid | 9009.4 | 20626 | 1619 | 15409 | 23780 | 4669 | 0.584684 |
| glycerol | 40938.2 | 75518 | 23188 | 78015 | 136588 | 45634 | 0.524748 |
| glycerol-3-galactoside | 1962.6 | 2355 | 1675 | 2809.333 | 4099 | 1540 | 0.6986 |
| glycine | 34846 | 100499 | 9946 | 12662 | 18261 | 8978 | 2.752014 |
| glycocyamine | 6163.2 | 17078 | 777 | 12856.83 | 26976 | 3860 | 0.479372 |
| glycolic acid | 1072.8 | 1623 | 206 | 1558.333 | 2368 | 768 | 0.688428 |
| hexadecane | 5326 | 11579 | 2105 | 2304.333 | 3666 | 1192 | 2.311298 |
| hexitol | 4650 | 6645 | 1046 | 5531.333 | 10566 | 2901 | 0.840665 |
| hexuronic acid | 2276 | 3102 | 1028 | 2170.667 | 2678 | 1648 | 1.048526 |
| hippuric acid | 53228.4 | 176676 | 19241 | 141722.8 | 242920 | 31514 | 0.375581 |
| hydroxylamine | 257379.8 | 474516 | 88417 | 115960 | 154609 | 92660 | 2.219557 |
| indole-3-acetate | 410.2 | 1088 | 112 | 1412.833 | 3376 | 363 | 0.290339 |
| indoxyl sulfate | 39197.6 | 74744 | 8163 | 42593.67 | 74252 | 7074 | 0.920268 |
| inosine | 375.4 | 947 | 84 | 350.1667 | 460 | 242 | 1.072061 |
| isocitric acid | 31693.4 | 49436 | 10172 | 44161.5 | 55474 | 33555 | 0.71767 |
| isohexonic acid | 8881 | 13185 | 513 | 17078.83 | 42027 | 861 | 0.52 |
| isomaltose | 1495.2 | 4080 | 418 | 1257.5 | 3344 | 480 | 1.189026 |
| isoribose | 2888.8 | 3684 | 1822 | 4301.833 | 5899 | 3291 | 0.671528 |
| isothreonic acid | 24474.6 | 34083 | 18174 | 27020.33 | 36258 | 9297 | 0.905785 |
| kynurenic acid | 553 | 852 | 305 | 899.8333 | 1130 | 579 | 0.614558 |
| lactic acid | 20891.4 | 60266 | 3846 | 2211.667 | 2972 | 1393 | 9.445998 |
| lysine | 1795.6 | 3624 | 92 | 8947.5 | 17950 | 256 | 0.200682 |
| lyxitol | 43421.8 | 48288 | 30229 | 68921.17 | 91577 | 52354 | 0.630021 |
| lyxose | 5311.6 | 18911 | 1097 | 3853 | 9104 | 2424 | 1.378562 |
| malic acid | 1892.8 | 6950 | 335 | 398.5 | 743 | 141 | 4.749812 |
| mannose | 3509.2 | 8293 | 2094 | 3261.667 | 8495 | 1821 | 1.075892 |
| mucic acid | 2189.6 | 2534 | 1811 | 4997.5 | 11764 | 2033 | 0.438139 |
| myo-inositol | 16141 | 24513 | 10262 | 18176.67 | 34737 | 9915 | 0.888007 |
| myristic acid | 520.6 | 1072 | 146 | 494.3333 | 546 | 367 | 1.053136 |
| N-acetylaspartic acid | 4991 | 16739 | 1082 | 9614.667 | 39250 | 1927 | 0.519103 |
| n-acetyl-d-hexosamine | 2825 | 5828 | 1130 | 3874.833 | 5441 | 2949 | 0.729064 |
| N-acetylglutamate | 621 | 1283 | 140 | 677.1667 | 1156 | 255 | 0.917056 |
| N-acetylmannosamine | 1533.4 | 2209 | 312 | 2036.333 | 3285 | 167 | 0.75302 |
| ornithine | 5004 | 11707 | 154 | 2845.333 | 5210 | 825 | 1.758669 |
| oxalic acid | 2002.8 | 2227 | 1432 | 2755.333 | 4442 | 1302 | 0.726881 |
| oxoproline | 66929 | 92099 | 39996 | 137876.8 | 326044 | 71684 | 0.485426 |
| palmitic acid | 22860.4 | 51404 | 12264 | 11560.33 | 14944 | 7695 | 1.977486 |
| pelargonic acid | 9267.6 | 19194 | 4277 | 3746.667 | 5476 | 2800 | 2.473559 |
| pentitol | 365.2 | 582 | 166 | 587.5 | 1461 | 80 | 0.621617 |
| phenaceturic acid | 158020.8 | 321965 | 93562 | 245175.2 | 298446 | 185225 | 0.644522 |
| phenol | 6777.4 | 17033 | 330 | 23829.33 | 42477 | 1390 | 0.284414 |
| phosphate | 866635 | 1104688 | 501482 | 749454 | 1397209 | 373315 | 1.156355 |
| pimelic acid | 1744.2 | 2887 | 1064 | 2521.667 | 3230 | 1629 | 0.691685 |
| pinitol | 27171 | 34278 | 10218 | 53952.5 | 85444 | 31561 | 0.50361 |
| propane-1,3-diol NIST | 7725 | 14715 | 2325 | 3950.5 | 5455 | 2733 | 1.955449 |
| pseudo uridine | 83432.2 | 158803 | 5641 | 168119.8 | 187497 | 151157 | 0.496266 |
| putrescine | 2340.2 | 4462 | 476 | 3468 | 4501 | 2183 | 0.674798 |
| pyruvic acid | 5213.8 | 10222 | 768 | 5252.333 | 7732 | 3131 | 0.992664 |
| quinic acid | 648.4 | 944 | 481 | 1034.833 | 1420 | 725 | 0.626574 |
| raffinose | 2886.6 | 3900 | 1439 | 7625.333 | 14675 | 2335 | 0.378554 |
| ribitol | 13601.4 | 42840 | 2754 | 18818.83 | 36434 | 9519 | 0.722755 |
| ribonic acid | 2793 | 6301 | 172 | 4314.167 | 7316 | 2606 | 0.647402 |
| ribose | 5631.6 | 8408 | 4475 | 6895.333 | 8739 | 5343 | 0.816726 |
| saccharic acid | 10841.8 | 13886 | 8720 | 20876.83 | 44917 | 10192 | 0.519322 |
| serine | 407.2 | 996 | 105 | 689.3333 | 1974 | 269 | 0.590716 |
| sorbitol | 12361 | 16569 | 6886 | 14407.67 | 29529 | 8494 | 0.857946 |
| stearic acid | 126977 | 312918 | 59058 | 68280.67 | 95190 | 46265 | 1.859633 |
| succinic acid | 743.2 | 1013 | 511 | 1361.667 | 4676 | 541 | 0.545802 |
| sucrose | 7002 | 13001 | 4113 | 11800.5 | 18130 | 2795 | 0.593365 |
| sulfuric acid | 30562.6 | 64456 | 2613 | 33828 | 54677 | 6394 | 0.90347 |
| tagatose | 519.2 | 845 | 267 | 728.6667 | 951 | 508 | 0.712534 |
| taurine | 13073 | 49458 | 206 | 53652.5 | 80588 | 12091 | 0.243661 |
| threitol | 6386.4 | 10639 | 3833 | 8596.333 | 10927 | 7188 | 0.742921 |
| threonic acid | 4396.6 | 4916 | 4008 | 6466.5 | 9759 | 4889 | 0.679904 |
| trehalose | 4855.8 | 5772 | 3889 | 7766.667 | 18721 | 4228 | 0.62521 |
| tryptophan | 1561.2 | 2428 | 1002 | 3915.667 | 8152 | 1902 | 0.398706 |
| tyrosine | 18392.2 | 25887 | 12662 | 46970.83 | 67566 | 32339 | 0.391566 |
| tyrosol | 1796.4 | 2917 | 469 | 2925.333 | 4797 | 1535 | 0.614084 |
| urea | 4197183 | 5810288 | 2070677 | 1149231 | 2109411 | 85079 | 3.652165 |
| uric acid | 10657.4 | 47272 | 275 | 24703.5 | 47340 | 12220 | 0.431413 |
| uridine | 1011 | 1639 | 352 | 2088.5 | 2849 | 1226 | 0.484079 |
| valine | 5667.4 | 12180 | 1060 | 2204.167 | 3664 | 870 | 2.571221 |
| vanillic acid | 1299.8 | 2474 | 695 | 1577.833 | 2212 | 1149 | 0.823788 |
| xylitol | 6458.4 | 10457 | 2698 | 13293.33 | 19483 | 9714 | 0.485838 |
| xylonic acid | 424 | 734 | 173 | 929.1667 | 1608 | 651 | 0.456323 |
| xylose | 20812 | 36032 | 13060 | 24786.33 | 34843 | 14364 | 0.839656 |
| xylulose NIST | 909 | 1569 | 253 | 1879.667 | 2718 | 1032 | 0.483596 |

| **Time 5** | **Average** | **Max** | **Min** | **Average** | **Max** | **Min** | **Ratio** |
| --- | --- | --- | --- | --- | --- | --- | --- |
| **Phenotype** | **M** | **M** | **M** | **C** | **C** | **C** | **M/C** |
| 3-(3-hydroxyphenyl)propionic acid | 34583.4 | 81070 | 2584 | 39735.5 | 89826 | 4948 | 0.87034 |
| 3-(4-hydroxyphenyl)propionic acid | 20740.2 | 34768 | 15464 | 40642 | 76961 | 17325 | 0.510314 |
| 3,4-dihydroxycinnamic acid | 785.6 | 1427 | 347 | 1524.5 | 2808 | 514 | 0.515316 |
| 3,4-dihydroxyhydrocinnamic acid NIST | 20581.4 | 60379 | 2808 | 24306.33 | 73331 | 1767 | 0.846751 |
| 3,4-dihydroxyphenylacetic acid | 5608.6 | 7537 | 1930 | 5752.667 | 7889 | 3125 | 0.974957 |
| 3-aminoisobutyric acid | 859.2 | 1988 | 205 | 19195.67 | 109879 | 336 | 0.04476 |
| 3-hydroxy-3-methylglutaric acid | 2406.2 | 3049 | 1982 | 1177 | 2821 | 332 | 2.04435 |
| 3-phosphoglycerate | 1834 | 3073 | 415 | 3296.333 | 9268 | 1303 | 0.556376 |
| 4-hydroxybenzoate | 860.8 | 1807 | 479 | 676.8333 | 904 | 513 | 1.271805 |
| 4-hydroxybutyric acid | 3274.2 | 4854 | 2267 | 2198.5 | 3575 | 1355 | 1.489288 |
| 4-hydroxycinnamic acid | 770.8 | 950 | 591 | 1128.333 | 1735 | 549 | 0.683131 |
| 4-hydroxyhippuric acid NIST | 6392 | 8487 | 4113 | 8212.167 | 10766 | 6521 | 0.778357 |
| 4-hydroxyphenylacetic acid | 46927.4 | 105169 | 6988 | 44289.17 | 65892 | 28757 | 1.059568 |
| 5-hydroxy-3-indoleacetic acid | 3110.6 | 3993 | 2063 | 4991.667 | 8158 | 2179 | 0.623159 |
| aconitic acid | 19901.8 | 24005 | 16940 | 22694 | 29012 | 15146 | 0.876963 |
| adenosine | 3185.4 | 7026 | 994 | 7994.333 | 17950 | 3256 | 0.398457 |
| alanine | 21944.4 | 27741 | 17749 | 25631.17 | 36797 | 12026 | 0.856161 |
| allantoic acid | 7506.4 | 14690 | 394 | 26986.67 | 72526 | 8827 | 0.278152 |
| alpha-ketoglutarate | 9885.2 | 17268 | 4353 | 3955.5 | 5957 | 2138 | 2.499103 |
| benzoic acid | 14431.8 | 21717 | 10495 | 12963.83 | 16359 | 7808 | 1.113236 |
| benzylalcohol | 16023.4 | 50868 | 1484 | 44628.5 | 113368 | 1579 | 0.35904 |
| beta-alanine | 8425.2 | 24126 | 381 | 9743.167 | 15761 | 2660 | 0.864729 |
| beta-gentiobiose | 8689 | 13121 | 2891 | 12574.5 | 19932 | 3663 | 0.691002 |
| catechol | 636.4 | 2279 | 37 | 1672.167 | 4196 | 6 | 0.380584 |
| citramalic acid | 1773.4 | 2196 | 1383 | 1237.667 | 1591 | 900 | 1.432858 |
| citric acid | 680344.4 | 1014236 | 334534 | 152519.7 | 331901 | 74338 | 4.460699 |
| citrulline | 3077.4 | 4530 | 1403 | 6919.333 | 12758 | 3708 | 0.444754 |
| conduritol-beta-expoxide | 4237.6 | 7888 | 2631 | 4484.5 | 7913 | 3162 | 0.944944 |
| creatinine | 33601.6 | 90584 | 4316 | 76127.5 | 182271 | 7945 | 0.441386 |
| deoxypentitol | 2317.4 | 2996 | 1103 | 3631 | 4839 | 2463 | 0.638226 |
| erythritol | 74198.2 | 84507 | 60025 | 79513.33 | 97179 | 64711 | 0.933154 |
| ferulic acid | 890.8 | 1502 | 346 | 1444 | 2865 | 416 | 0.616898 |
| fructose | 12934.4 | 19382 | 8227 | 15575.33 | 23522 | 8542 | 0.830441 |
| fucose | 56549.6 | 72867 | 30980 | 78991 | 92748 | 55600 | 0.715899 |
| galactinol | 4720.8 | 11638 | 1188 | 14367.33 | 35828 | 3652 | 0.328579 |
| galactonic acid | 1436.2 | 2095 | 891 | 3596.333 | 4773 | 1833 | 0.399351 |
| gluconic acid | 2913.6 | 3859 | 1059 | 3888.667 | 5580 | 2815 | 0.749254 |
| glyceric acid | 8137.8 | 17010 | 3456 | 16383.5 | 32664 | 5752 | 0.496707 |
| glycerol | 51021 | 71274 | 33580 | 48320 | 89475 | 29431 | 1.055898 |
| glycerol-3-galactoside | 2337 | 2747 | 1973 | 3331.667 | 3889 | 2582 | 0.701451 |
| glycine | 14978.4 | 20347 | 10667 | 14939.5 | 18342 | 12360 | 1.002604 |
| glycocyamine | 4978 | 9672 | 480 | 16911.33 | 29729 | 1789 | 0.294359 |
| glycolic acid | 1372 | 1601 | 1188 | 1738.333 | 2352 | 856 | 0.789262 |
| hexadecane | 4740 | 8474 | 2683 | 2894.667 | 5074 | 2098 | 1.637494 |
| hexitol | 5473.8 | 10186 | 3091 | 5275 | 7652 | 3861 | 1.037687 |
| hexuronic acid | 1549 | 2507 | 510 | 2232 | 4086 | 487 | 0.693996 |
| hippuric acid | 33355.6 | 101938 | 12106 | 61070.5 | 134451 | 12283 | 0.546182 |
| hydroxylamine | 160280 | 239668 | 106694 | 143814.8 | 180398 | 102835 | 1.114489 |
| indole-3-acetate | 1029.4 | 1404 | 668 | 1704.833 | 3896 | 257 | 0.603813 |
| indoxyl sulfate | 37927.6 | 76351 | 14432 | 40863.67 | 72323 | 11210 | 0.92815 |
| inosine | 516 | 1052 | 254 | 501 | 908 | 167 | 1.02994 |
| isocitric acid | 47616 | 53082 | 43673 | 46816.5 | 61774 | 31995 | 1.017077 |
| isohexonic acid | 11252.6 | 16097 | 5727 | 16717.67 | 26825 | 9579 | 0.673096 |
| isomaltose | 1519 | 2804 | 725 | 873.1667 | 1667 | 161 | 1.739645 |
| isoribose | 3836.2 | 4537 | 3150 | 5856.167 | 6907 | 2441 | 0.65507 |
| isothreonic acid | 27533.2 | 30835 | 22895 | 31820 | 42357 | 22783 | 0.86528 |
| kynurenic acid | 489.8 | 628 | 420 | 1052.833 | 1517 | 637 | 0.465221 |
| lactic acid | 6156.4 | 16692 | 2300 | 3043.5 | 3891 | 1523 | 2.022803 |
| lysine | 2841.8 | 6795 | 179 | 14660.67 | 25178 | 260 | 0.193838 |
| lyxitol | 44520.8 | 52199 | 36215 | 69324.67 | 92237 | 42076 | 0.642207 |
| lyxose | 2945.4 | 3933 | 2158 | 3125.167 | 4222 | 2045 | 0.942478 |
| malic acid | 758 | 1435 | 239 | 212.6667 | 336 | 121 | 3.564263 |
| mannose | 3089.4 | 5236 | 1447 | 3556.167 | 5939 | 1510 | 0.868744 |
| mucic acid | 1830 | 3021 | 405 | 4098 | 6862 | 2023 | 0.446559 |
| myo-inositol | 14408.8 | 20097 | 2135 | 18400 | 24538 | 11034 | 0.783087 |
| myristic acid | 640.6 | 784 | 498 | 600.6667 | 919 | 422 | 1.066482 |
| N-acetylaspartic acid | 8363 | 17248 | 2832 | 12166.17 | 60766 | 1585 | 0.687398 |
| n-acetyl-d-hexosamine | 3636.8 | 3915 | 3180 | 4573 | 6095 | 2467 | 0.795277 |
| N-acetylglutamate | 498 | 645 | 287 | 662 | 970 | 463 | 0.752266 |
| N-acetylmannosamine | 2191.8 | 2850 | 1845 | 2730.167 | 4844 | 1882 | 0.802808 |
| ornithine | 5928.4 | 8823 | 2610 | 3993.833 | 5150 | 1453 | 1.484388 |
| oxalic acid | 2473.8 | 2951 | 1972 | 3286.5 | 4606 | 2687 | 0.752716 |
| oxoproline | 93162.6 | 130934 | 62086 | 121696.8 | 186403 | 76634 | 0.76553 |
| palmitic acid | 14539.6 | 21907 | 6769 | 12820.5 | 17316 | 9313 | 1.13409 |
| pelargonic acid | 5704 | 8435 | 2697 | 4712.167 | 7153 | 3039 | 1.210484 |
| pentitol | 636 | 939 | 461 | 959.6667 | 1470 | 520 | 0.66273 |
| phenaceturic acid | 205224.2 | 330187 | 119922 | 252089.8 | 383174 | 153666 | 0.814092 |
| phenol | 4098.8 | 10639 | 754 | 13964.67 | 39255 | 2966 | 0.293512 |
| phosphate | 807376.4 | 1002230 | 673562 | 647028.7 | 1549032 | 136123 | 1.247822 |
| pimelic acid | 1402.6 | 1987 | 796 | 2687.167 | 4023 | 1752 | 0.521962 |
| pinitol | 30595.8 | 42588 | 17263 | 47518.83 | 70727 | 29308 | 0.643867 |
| propane-1,3-diol NIST | 5367 | 6810 | 3858 | 4975.667 | 7172 | 2212 | 1.078649 |
| pseudo uridine | 98092 | 134441 | 41743 | 194827 | 251176 | 107469 | 0.503483 |
| putrescine | 2645.6 | 5165 | 361 | 4421.333 | 6132 | 2209 | 0.598372 |
| pyruvic acid | 7802.4 | 17047 | 4045 | 8667.333 | 15825 | 3769 | 0.900208 |
| quinic acid | 947.4 | 1200 | 426 | 1061.667 | 1730 | 278 | 0.89237 |
| raffinose | 3509.2 | 6269 | 814 | 6086.667 | 12853 | 718 | 0.576539 |
| ribitol | 13570.6 | 47495 | 3855 | 12400.83 | 17940 | 7217 | 1.09433 |
| ribonic acid | 3469.2 | 4559 | 2902 | 3759.833 | 6433 | 607 | 0.9227 |
| ribose | 5985.6 | 8635 | 703 | 7194.167 | 13066 | 1666 | 0.832007 |
| saccharic acid | 11602.8 | 16623 | 6007 | 17073 | 28166 | 9339 | 0.679599 |
| serine | 383.6 | 691 | 244 | 883.3333 | 2034 | 307 | 0.434264 |
| sorbitol | 17369.4 | 24282 | 8781 | 14643.33 | 25560 | 7089 | 1.186164 |
| stearic acid | 80805.2 | 114944 | 37679 | 69137.83 | 88660 | 50043 | 1.168755 |
| succinic acid | 1116.8 | 1927 | 549 | 1235.167 | 4972 | 291 | 0.904169 |
| sucrose | 8631.8 | 16332 | 1739 | 8193 | 15733 | 900 | 1.053558 |
| sulfuric acid | 77222.8 | 158793 | 32565 | 44257.17 | 129666 | 3241 | 1.744865 |
| tagatose | 573.8 | 681 | 492 | 786.3333 | 989 | 643 | 0.729716 |
| taurine | 48041.2 | 130725 | 67 | 221184 | 328263 | 84793 | 0.2172 |
| threitol | 7435.2 | 7893 | 6927 | 8832.667 | 10248 | 7541 | 0.841784 |
| threonic acid | 4572.2 | 5648 | 3824 | 6557.833 | 10380 | 4351 | 0.697212 |
| trehalose | 5550 | 7410 | 2046 | 6587.667 | 10946 | 1954 | 0.842483 |
| tryptophan | 1828.4 | 2762 | 874 | 4740.833 | 14161 | 1322 | 0.385671 |
| tyrosine | 31056.2 | 36016 | 27167 | 53870.67 | 63563 | 42199 | 0.576496 |
| tyrosol | 2479 | 4418 | 672 | 2355.5 | 3073 | 744 | 1.05243 |
| urea | 3347697 | 5352570 | 267617 | 1751767 | 5146029 | 138791 | 1.911041 |
| uric acid | 12322 | 18160 | 1770 | 26750.83 | 49651 | 5323 | 0.460621 |
| uridine | 1720.2 | 2283 | 899 | 2179.333 | 3450 | 1040 | 0.789324 |
| valine | 2588 | 3378 | 1427 | 2733.833 | 4568 | 951 | 0.946656 |
| vanillic acid | 1452.8 | 2142 | 555 | 1886.833 | 2616 | 1209 | 0.769967 |
| xylitol | 10542.2 | 15323 | 6402 | 16220.83 | 19386 | 8297 | 0.649917 |
| xylonic acid | 458.2 | 721 | 342 | 882.5 | 1299 | 296 | 0.519207 |
| xylose | 35249.6 | 44554 | 24659 | 33816.33 | 49727 | 26787 | 1.042384 |
| xylulose NIST | 1791.6 | 2510 | 1022 | 2654.833 | 3524 | 968 | 0.674845 |
